# Candidate genes and functional noncoding variants identified in a canine model of obsessive-compulsive disorder

**DOI:** 10.1186/gb-2014-15-3-r25

**Published:** 2014-03-14

**Authors:** Ruqi Tang, Hyun Ji Noh, Dongqing Wang, Snaevar Sigurdsson, Ross Swofford, Michele Perloski, Margaret Duxbury, Edward E Patterson, Julie Albright, Marta Castelhano, Adam Auton, Adam R Boyko, Guoping Feng, Kerstin Lindblad-Toh, Elinor K Karlsson

**Affiliations:** 1 Broad Institute of MIT and Harvard, 7 Cambridge Center, Cambridge, MA 02142, USA; 2 McGovern Institute for Brain Research and Department of Brain and Cognitive Sciences, Massachusetts Institute of Technology, Cambridge, MA 02139, USA; 3 Renji Hospital, School of Medicine, Shanghai Jiao Tong University, Shanghai 200127, China; 4 College of Veterinary Medicine, University of Minnesota, St. Paul, MN 55108, USA; 5 Department of Clinical Sciences, College of Veterinary Medicine, Cornell University, Ithaca, NY 14853, USA; 6 Department of Genetics, Albert Einstein College of Medicine, 1301 Morris Park Avenue, Van Etten B06, Bronx, NY 10461, USA; 7 Department of Biomedical Sciences, Cornell University, Ithaca, NY 14853, USA; 8 Science for Life Laboratory, Department of Medical Biochemistry and Microbiology, Uppsala University, Uppsala 75237, Sweden; 9 Center for Systems Biology, Department of Organismic and Evolutionary Biology, Harvard University, Cambridge, MA 02138, USA

## Abstract

**Background:**

Obsessive-compulsive disorder (OCD), a severe mental disease manifested in time-consuming repetition of behaviors, affects 1 to 3% of the human population. While highly heritable, complex genetics has hampered attempts to elucidate OCD etiology. Dogs suffer from naturally occurring compulsive disorders that closely model human OCD, manifested as an excessive repetition of normal canine behaviors that only partially responds to drug therapy. The limited diversity within dog breeds makes identifying underlying genetic factors easier.

**Results:**

We use genome-wide association of 87 Doberman Pinscher cases and 63 controls to identify genomic loci associated with OCD and sequence these regions in 8 affected dogs from high-risk breeds and 8 breed-matched controls. We find 119 variants in evolutionarily conserved sites that are specific to dogs with OCD. These case-only variants are significantly more common in high OCD risk breeds compared to breeds with no known psychiatric problems. Four genes, all with synaptic function, have the most case-only variation: neuronal cadherin (*CDH2*), catenin alpha2 (*CTNNA2*), ataxin-1 (*ATXN1*), and plasma glutamate carboxypeptidase (*PGCP*). In the 2 Mb gene desert between the cadherin genes *CDH2* and *DSC3,* we find two different variants found only in dogs with OCD that disrupt the same highly conserved regulatory element. These variants cause significant changes in gene expression in a human neuroblastoma cell line, likely due to disrupted transcription factor binding.

**Conclusions:**

The limited genetic diversity of dog breeds facilitates identification of genes, functional variants and regulatory pathways underlying complex psychiatric disorders that are mechanistically similar in dogs and humans.

## Background

Obsessive compulsive disorder (OCD) is a common (1 to 3% of the population) and debilitating neuropsychiatric disorder characterized by persistent intrusive thoughts and time-consuming repetitive behaviors [1]. Twin studies show OCD is very heritable (approximately 45 to 65% genetic influences for early onset OCD), but the underlying genetics is complex [2,3]. More than 80 candidate gene studies of OCD and a recent genome-wide association study (GWAS) yielded no significant, replicable associations [4]. The most strongly associated genes in the OCD GWAS implicate disrupted glutamatergic neurotransmission and signaling in disease pathogenesis [4].

Artificial mouse models have proven more effective for elucidating the neural pathways underlying OCD-like behaviors. Mice lacking *Sapap3*, which encodes a postsynaptic scaffolding protein found at glutamatergic synapses, exhibited excessive grooming and increased anxiety, symptoms alleviated by treatment with selective serotonin reuptake inhibitors, the same drug frequently used to treat OCD patients [5]. Optogenetic stimulation of the orbitofrontal cortex region affected by the *Sapap3* mutation reversed defective neural activity and suppressed compulsive behavior [6]. Resequencing of exons of *DLGAP3* (the human *SAPAP3* gene) revealed excessive rare non-synonymous variants in human OCD and trichotillomania individuals [7].

Canine OCD is a naturally occurring model for human OCD that is genetically more complex than induced animal models [8]. Phenotypically, canine and human OCD are remarkably similar. Canine compulsive disorder manifests as repetition of normal canine behaviors such as grooming (lick dermatitis), predatory behavior (tail chasing) and suckling (flank and blanket sucking). Just as in human patients, approximately 50% of dogs respond to the treatment with selective serotonin reuptake inhibitors or clomipramine [9]. Particular dog breeds (genetically isolated populations) have exceptionally high rates of OCD, including Doberman Pinschers (DPs), bull terriers and German shepherds [10-12]. The high disease rates and rather limited genetic diversity of dog breeds suggests that OCD in these populations, while multi-genic, may be less complex than in humans, facilitating genetic mapping and functional testing of associated variants [13,14].

In an earlier GWAS of canine OCD, we associated *CDH2*, a neural cadherin gene involved in synaptic plasticity, with OCD in DPs [14]. Here, we use a more powerful algorithm, MAGIC [15], to reanalyze the data from this study and identify new OCD-associated regions. These regions are enriched for genes involved in synapse formation and function, as are regions with patterns of reduced variation consistent with artificial selection. We sequence the top candidate regions, 5.8 Mb in total, and find that four genes, all with synaptic function, are enriched for case-specific variants: neuronal-cadherin (*CDH2*), catenin alpha2 (*CTNNA2*), ataxin-1 (*ATXN1*), and plasma glutamate carboxypeptidase (*PGCP*). Furthermore, two intergenic mutations between the cadherin genes *CDH2* and desmocollin 3 (*DSC3*) disrupt a non-coding regulatory element and alter gene expression in a human neuroblastoma cell line. Our results implicate abnormal synapse formation and plasticity in OCD, and point to disrupted expression of neural cadherin genes as one possible cause.

## Results

### Genome-wide association studies and homozygosity mapping

**Figure 1:**
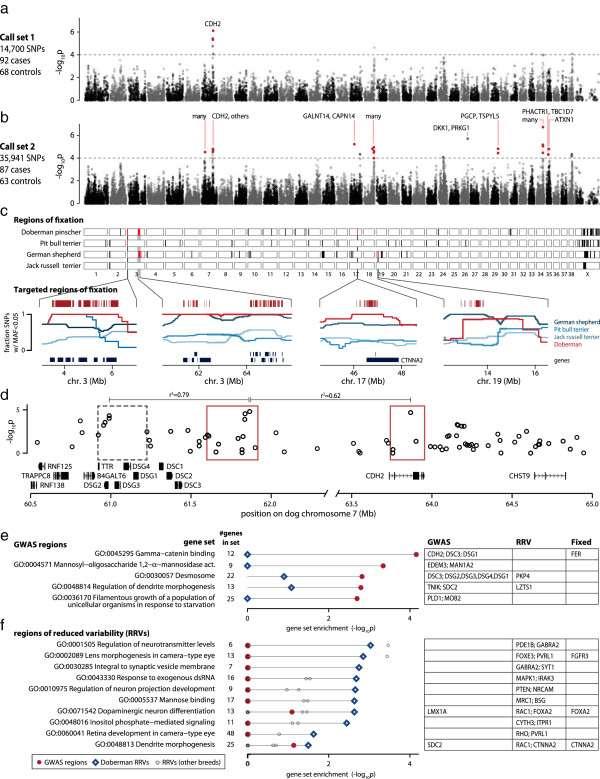
**Associated and fixed regions in Doberman Pinschers are enriched for brain-related pathways. (a)** The original GWAS dataset showed a single peak of association at *CDH2*[14]. **(b)** Recalling with MAGIC yielded a 2.4-fold denser SNP dataset and allowed us to define 17 distinct regions of association with *P* < 0.0001 using linkage disequilibrium (LD) clumping (Figure S1 in Additional file 1), a subset of which were targeted for sequencing (red dots, genes labeled above peak). **(c)** In four breeds with high rates of OCD, we identified regions of fixation (black boxes), a subset of which we targeted for sequencing (red boxes). Sequenced regions were selected because they were large and overlapping between breeds (Table S1 in Additional file 1). **(d)** LD clumping identified three distinct regions of association on chromosome 7 (boxes, with targeted regions in red). **(e)** The top Gene Ontology gene sets enriched in the GWAS regions. **(f)** GO gene sets enriched in Doberman Pinscher regions of reduced variability (RRVs) but not in 24 other breeds (grey circles, most at 0).

Using the raw data (included in Gene Expression Omnibus accession numbers GSE53488 and GSE53577) from the GWAS by Dr Dodman and collaborators [14], which included 92 DP cases and 68 DP controls extensively phenotyped for canine OCD, we reanalyzed the Affymetrix genotype intensity data with a new calling algorithm, MAGIC [15]. MAGIC relaxes certain assumptions used in other callers, such as Hardy-Weinberg equilibrium in genotype clusters, to dramatically improve the accuracy of genotypes called from Affymetix v2 Canine GeneChip data. This yielded a 2.4-fold denser SNP map for association mapping (55,651 SNPs; 35,941 SNPs with minor allele frequency (MAF) >0.05) but a slightly smaller sample size, with 87 cases and 63 controls passing MAGIC quality filters (compared to our original dataset of 14,700 SNPs with MAF > 0.05 in 92 cases and 68 controls; Figure 1a,b). The increased density allowed us to identify 13 new candidate OCD-associated regions (*P* < 0.0001) in addition to the original chromosome 7 locus in *CDH2* (Table S1 in Additional file 1). We estimate that this dataset explains 0.56 ± 0.18 of phenotype variance [16].

We tested all Gene Ontology (GO) gene sets with 5 to 1,000 genes (5,206 sets) for enrichment in the new GWAS regions using INRICH, a permutation based software that rigorously controls for region size, SNP density, and gene size and gene number [17]. Overall, we observe an excess of sets with *P* < 0.01 (25 sets, *P* = 0.03; Figure 1e). The top set ‘GO:0045295 Gamma catenin binding’ is significant even after stringent correction for the number of gene sets tested (*P* = 5.9 × 10^-5^*, P*_*corrected*_ = 0.05) and includes genes under each of three peaks of association spanning approximately 3 Mb on chromosome 7: *CDH2*, *DSC3* and *DSG1* (Figure 1d,e; Table S2 in Additional file 1). The GWAS regions also include two of 13 genes in ‘GO:0048814 Regulation of dendrite morphogenesis’ (*P* = 0.002): the calcium binding synaptogenesis gene *SDC2* and the postsynaptic density protein gene *TNIK*, which encodes a serine-threonine kinase involved in AMPA receptor trafficking and synaptic function [18,19].

The DP breed, like all dog breeds, was created through population bottlenecks and artificial selection for morphological and behavioral traits, potentially driving some OCD risk alleles to very high frequency and thus undetectable by GWAS. Consistent with this hypothesis, we find functional connections between associated genes and genes in the 13 largest autosomal regions of fixation in the DP breed (25.7 Mb in total; Table S3 in Additional file 1). For example, the tyrosine kinase FER mediates cross-talk between CDH2 and integrins [20], and depletion of presynaptic FER inhibits synaptic formation and transmission [21]. CTNNA2 interacts with CDH2 to regulate the stability of synaptic cell junctions [22]. While most fixed regions contain many genes, making it difficult to identify top candidates, several contain just one gene, including the neuronal protein gene *LINGO2* and the synaptic-2 like glycoprotein gene *TECRL*.

We also identified 128 regions of unusually low variability in the DP breed compared to 24 other dog breeds (23.73 Mb; Table S4 in Additional file 1) [23]. When we test these regions of reduced variability (RRVs) for gene set enrichment in the entire GO catalog, as described above, 10 GO terms are more enriched in DP RRVs than any other breed (Figure 1f). Half of these have clear relevance to brain function, including regulation of neurotransmitters, neural projection, and dendrite morphogenesis. We also see enrichment for mannose binding-related genes, echoing the strong enrichment in GWAS regions for alpha-mannosidase activity. Mannose structures are concentrated at excitatory synapses, including glutamate receptors [24,25].

### Targeted sequencing

**Figure 2:**
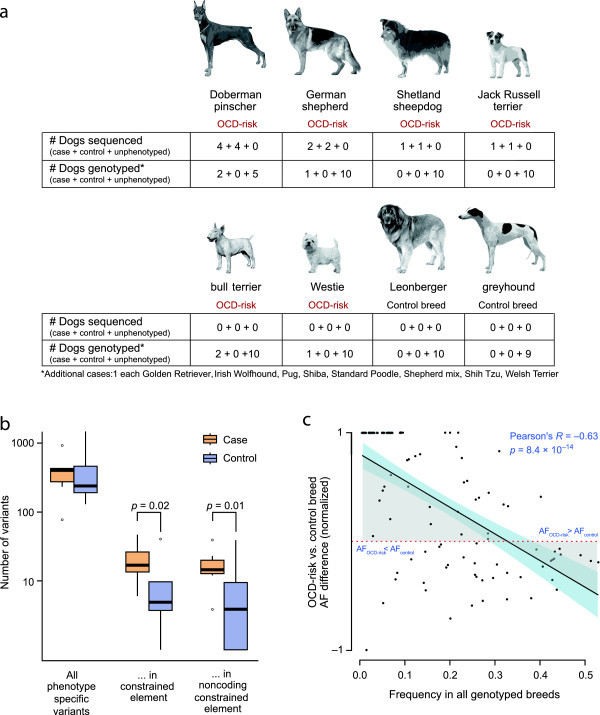
**Targeted sequencing identifies case-only variants that alter constrained elements and are more common in high OCD risk breeds. (a)** We performed targeted sequencing of a small number of cases and controls from four breeds (row 1) and subsequently genotyped the top candidate variants in a larger panel of dogs from those four breeds as well as two more high ‘OCD-risk’ breeds and two low risk ‘control breeds’ (row 2). **(b)** Across all variants identified in the sequencing data, the number of case-only (orange box) and control-only (purple box) variants is similar, but constrained elements are enriched for case-only variants. Boxes mark the 25th to 75th percentile across dogs, with the median shown as a thick line, and whiskers extending to values within 1.5 times the difference between the 25th to 75th percentiles. Outliers are marked with circles. **(c)** Case-only variants have higher frequency in OCD-risk breeds and lower frequency across all genotyped breeds. The x-axis represents allele frequencies across all genotyped dogs. The y-axis represents normalized allele frequency (AF) differences between OCD-risk and control breeds ([AF_OCD-risk_ - AF_control_]/[AF_OCD-risk_ + AF_control_]). The straight downward line represents the linear model for the data points. The blue shade shows the 95% confidence interval for this model. Area under the curve (shaded in grey) is notably larger in AF_OCD-risk_ > AF_control_ than in AF_OCD-risk_ < AF_control_, showing that case-only variants are more common in OCD-risk breeds than in control breeds.

We designed a sequencing array (Tables S5 and S6 in Additional file 1) that targeted nine of the top GWAS regions, including the *CDH2* locus (3.9 Mb; Figure S1 in Additional file 1) as well as genes and conserved elements within the five largest DP fixed regions (Table S3 in Additional file 1). We focused on fixed regions (totaling 1.8 Mb) that were also fixed in two other OCD affected breeds, German shepherds (LUPA reference panel [26]) and bull terriers (20 dogs) (Figure 1c). We sequenced eight cases and eight matched controls from breeds at high risk for OCD, including eight DP, four German shepherds, two Shetland sheepdogs and two Jack Russell terriers (Figure 2a). We selected DPs based on their genotype for the CDH2 risk haplotype [14] (two homozygous cases, two heterozygous cases, and four controls without the risk haplotype). We captured 92% of the target regions at >20× coverage, with 76× mean read depth coverage per sample (Table S7 in Additional file 1). In total, we detected 24,930 high-quality SNPs, 7,645 short INDELs, and 173 deletions, with high concordance to the SNP array data (median 99.5% for approximately 390 SNPs tested per sample; Table S8 in Additional file 1).

### Case-only variant discovery from sequence data

With our small sample size (eight cases and eight controls from four different breeds), we did not expect to have sufficient power to detect statistically significant allelic associations with OCD. Instead, we focused on variants seen only in OCD cases (‘case-only variants’) as the strongest causal candidates. Of 32,575 variants, 2,291 variants are case-only (2,002 SNPs and 289 INDELs; 80 to 966 per dog), while 3,116 variants are specific to control dogs (‘control-only variants’; 2,698 SNPs and 418 INDELs; 156 to 1,476 per dog) (Table 1; Table S9 in Additional file 1). While there is no significant difference between the total number of case- and control-only variants (Wilcoxon test *P* = 0.63; Figure 2b), case dogs have a significantly greater number in evolutionarily constrained elements (median 15 versus 4, Wilcoxon test *P* = 0.02; Materials and methods; Figure 2b; Table S9 in Additional file 1). Excluding coding variants increases the difference further (median 15 versus 3, Wilcoxon test *P* = 0.01), suggesting that the excess of case-only functional variants may be due largely to noncoding variation.

**Table 1:** Sequence variants identified by targeted resequencing of 5.8 Mb in eight cases and eight controls

**Annotations**	**All 16 dogs**	**Variants in cases**	**Variants in controls**	**Case-only variants**	**Control-only variants**
All variants	32,575	29,425	30,253	2,291	3,116
Missense mutations	71	64	61	6	7
Nonsense mutations	0	0	0	0	0
Frame-shift mutations	2	3	3	0	0
Silent coding variants	108	97	89	18	11
UTR variants	22	16	22	0	6
Essential splice site	1	1	1	0	0
Conserved sites^a^	1,024	930	908	119	91

^a^Conserved sites determined by 29 mammals sequence data set [27].

### Genotyping case-only variants in independent samples

We genotyped 114 case-only, evolutionarily constrained variants in an independent set of dogs from breeds with high rates of OCD (‘OCD-risk breeds’; 69 dogs) and breeds with normal rates of OCD and other psychiatric disorders (‘control breeds’; 19 dogs). Except for 14 cases from OCD-risk breeds, we have no individual OCD phenotype information for these dogs (Figure 2a). We find that the case-only variants identified in the sequence data are significantly more common in OCD-risk breeds, with median frequency (F_OCD_) of 0.17, than in control breeds, where the median frequency (F_control_) is 0.05 (Wilcoxon test *P* = 0.045; Table S10 in Additional file 1). The median frequency increases to 0.20 when only phenotyped cases are considered (Wilcoxon test *P* = 0.015, comparison with F_control_). We also observe an inverse correlation between the frequency difference between OCD-risk and control breeds and the frequency across all genotyped dogs (Pearson’s R = -0.63, *P* = 8.4 × 10^-14^; Figure 2c). Thus, the variants most enriched in OCD-risk breeds are otherwise rare, potentially due to either positive selective pressure in OCD-risk breeds or negative selection in the control breeds. While this suggests an association with OCD, we note that other traits may also systematically differ between the two breed groups.

### Gene-based analysis

**Figure 3:**
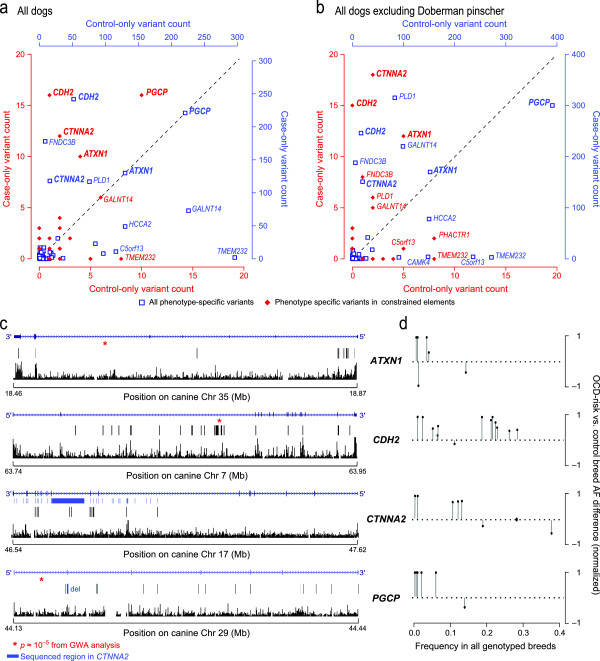
**Gene-based analysis identifies four top genes enriched for case-only variants in constrained regions. (a)** Genes are plotted according to the number of case-only (y-axis) and control-only (x-axis) variants within a gene and its 5 kb flanking regions from our sequence data. Blue squares denote all the variants and the corresponding axes are shown in blue; red diamonds denote variants in constrained elements only and the corresponding axes are shown in red. Genes that are plotted above the identity line harbor more case-only than control-only variants. **(b)** A similar analysis excluding DP, the breed used to identify genes for sequencing, shows that the enrichment pattern persists for several genes. **(c)** The case-only variants in constrained elements, when plotted with gene structure and evolutionary conservation, show clustering in *ATXN1* (5’ end). Dimmed bars represent canine variants that failed to lift over onto hg19. The conservation track shows a measure of evolutionary conservation in dog, human, mouse and rat [29]. **(d)** In each gene, SNPs with the greatest risk allele frequency (AF) difference between OCD-risk and control breeds (y-axis) tend to have lower frequency across all genotyped breeds (y-axis). SNPs are shown as solid circles with vertical lines.

We identified genes enriched with case-only variants using a gene-based analysis method that accounts for multiple independent variants within a gene and greatly increases power for identifying disease-associated genes [28]. Four genes have an excess of case-only variation in evolutionarily constrained elements, even after correcting for gene size: *ATXN1*, *CDH2*, *CTNNA2*, and *PGCP* (10, 16, 12, and 16 case-only variants, respectively; Figure 3a; Text S1 and Table S11 in Additional file 1). Because the sequenced DPs were selected based on their haplotype at *CDH2*, we confirmed that the case-only enrichment at *CDH2* persists even when DPs are excluded (Figure 3b). RNA-Seq data show all four genes are expressed in the dog brain (KL-T, unpublished observations).

Three of the four candidate genes, *CDH2*, *PGCP* and *ATXN1*, are associated with OCD in the DP GWAS study (chr7:63867472, *P* = 2.1 × 10^-5^; chr29:44152594, *P* = 1.5 × 10^-5^; chr35:18565131, P = 1.6 × 10^-5^, respectively), while the fourth, *CTNNA2*, falls in a large region of fixation (900 kb) in the DP breed (Figure 3c). In our genotyping dataset, the case-only variants in these four genes are more common in OCD-risk breeds (F_OCD_ = 0.08 versus F_control_ = 0.026, Wilcoxon test *P* = 2.95 × 10^-4^; Figure 3d), particularly in *CDH2* (F_OCD_ = 0.23 versus F_control_ = 0.027, *P* = 0.001) and in *PGCP* (F_OCD_ = 0.014 versus F_control_ = 0.0, *P* = 0.047). We see a similar, though weaker, pattern in *ATXN1* (F_OCD_ = 0.022 versus F_control_ = 0.0, *P* = 0.3) and *CTNNA2* (F_OCD_ = 0.185 versus F_control_ = 0.026, *P* = 0.13). In *CTNNA2*, the difference is clearer (*P* = 0.054) if only variants with frequency <0.20 are considered.

Of the 40 variants genotyped in these four genes, seven overlap chromatin marks, potentially indicating regulatory function. Four variants in *CDH2* overlap H3K27Ac histone marks and/or DNase1 hypersensitivity clusters. Three of these (chr7:63845160, chr7:63852056, and chr7:63832008) are observed in OCD-risk breeds, at frequencies of 0.435, 0.050, and 0.022, respectively, and never seen in control breeds. The fourth variant (chr7:63806661) is four-fold more common in OCD-risk breeds (frequency = 0.11 versus 0.026 in control breeds). Three variants in *ATXN1* alter regions transcribed in the dog brain (KL-T, unpublished RNA-Seq data), including a putative enhancer variant not seen in the control breeds (chr35:18850625, OCD-risk breed frequency = 0.014). These variants, which lie in genes enriched for case-only variants, are overrepresented in cases, and alter putative regulatory elements, are strong candidates for further functional elucidation.

### Single variant analysis

We next sought to identify the top candidate functional variants in the sequencing data. We first looked for coding variants found exclusively in cases. Most were missense mutations disrupting genes with little known relevance to brain functions (Table S12 and Text S2 in Additional file 1). More intriguing, one of our two Jack Russell terrier cases has a 1.2 kb deletion (chr29:44178339–44179516; Figure S2 in Additional file 1) overlapping exon 2 of the gene *PGCP*, causing a frameshift and loss of 70 amino acids from the protein. *PGCP* is one of the four genes enriched for case-only variants, and, while none of the DP cases has this particular deletion, a nearby SNP is among the most strongly associated in the GWAS (chr29:44152594, *P* = 1.5 × 10^-5^; Figure 1; Figure S3 in Additional file 1). Using quantitative PCR (qPCR), we validated the deletion in the Jack Russell terrier cases and tested 74 dogs from OCD-risk breeds (including 10 unphenotyped Jack Russell terriers and 14 dogs from several breeds diagnosed with OCD) and 20 dogs from control breeds. We found the deletion in three Jack Russell terriers and in one Welsh terrier with OCD, and in none of the control breed dogs, suggesting it is associated with increased risk of OCD in multiple breeds.

**Figure 4:**
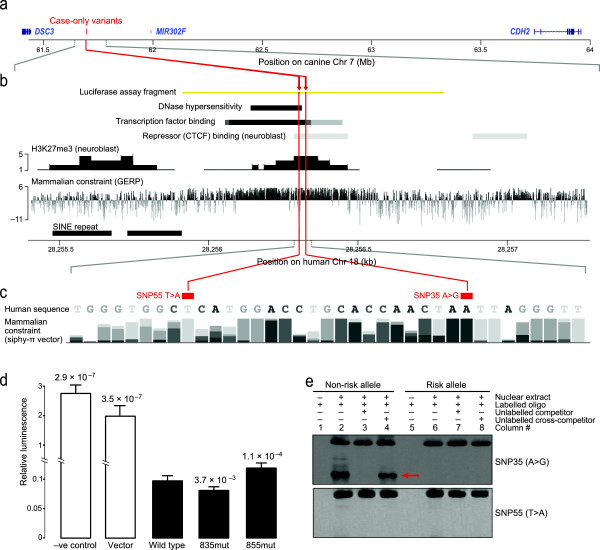
**Two intergenic case-only variants disrupt a repressor element and change gene expression *in vitro*. (a)** The two case-only variants are just 20 bases apart in a 2.5 Mb gene desert between *DSC3* and *CDH2* on canine chromosome 7. **(b)** The syntenic region of human chromosome 18 shows markers of DNase hypersensitivity, transcription factor binding, repressor binding, histone methylation [30], and mammalian constraint [31]. **(c)** Both variants, SNP55 and SNP35, alter bases that are highly constrained across mammals, SNP55 to an A and SNP35 to a T. Compositions of the four bases for each position in the 29 mammals comparison [27] are shown with different grey scale and height of bars (the higher the more conserved in multiple species). **(d)** The wild-type regulatory sequence represses luciferase reporter expression in SK-N-BE(2) neuroblastoma cells. Both variants significantly change the extent of repression relative to wild type, with SNP35-G more repressive and SNP55-A less repressive. The firefly luciferase expression in the test plasmids were normalized against the co-transfected Renilla luciferase expression in pGL4.73. The *P*-value of the significance of the change relative to wild type is shown above each bar, with vertical lines showing standard error of the mean. **(e)** An electrophoretic mobility shift assay testing the wild-type alleles (lanes 1 to 4) and the OCD-risk alleles (lanes 5 to 8) of SNP35 (top gel) and SNP55 (bottom gel) show that nuclear protein binding (red arrow) to the SNP35 locus is disrupted by the risk allele. Nuclear extract was derived from SK-N-BE(2) cells. We used a 200-fold molar excess of competitor where appropriate.

We next looked for non-coding variants seen only in cases, focusing on 15 seen in more than one DP case. All but two are near the GWAS peak in intron 2 of *CDH2* (chr7:63867472, *P* = 2.1 × 10^-5^), reflecting the selection of DP dogs for sequencing based on their genotype at this locus. None of the 13 is obviously functional based on evolutionary constraint and histone marks. The other two variants are more interesting, changing a conserved region approximately 172 kb away from an associated GWAS SNP (chr7:61865715, *P* = 1.6 × 10^-5^), in the gene desert between the cadherin genes *CDH2* and *DSC3* (Figure 4a). The first SNP (chr7:61693835, T changed to C; SNP35 T > C) is exclusively found in three of four sequenced DP cases and showed the overall DP breed frequency of approximately 0.30 in our genotyping data set. The second SNP, a private variant in the fourth DP case (chr7:61693855; SNP55 A > T), is just 20 bases away and alters the same highly conserved region (Figure 4b,c).

### Functional assessment of candidate variants

Because the region altered by SNP35 and SNP55 showed evidence of regulatory function (Figure 4b), we tested whether the risk alleles disrupt gene expression using a luciferase reporter assay. Including the wild-type region in the reporter construct lowers expression 14- to 20-fold in human neuroblastoma SK-N-BE(2) cells (vector versus wild type, *t*-test Bonferroni corrected *P* = 3.5 × 10^-7^*;* negative control versus wild type, *t*-test Bonferroni corrected *P* = 2.9 × 10^-7^*;* Figure 4d). Adding the SNP55 risk variant to the construct, however, significantly increases expression relative to the wild-type version, suggesting the regulatory element no longer functions normally (1.6-fold change, paired *t*-test Bonferroni corrected *P* = 1.1 × 10^-4^; Figure 4d). Curiously, the SNP35 risk allele has the opposite effect, repressing expression even further (0.9-fold change, paired *t*-test Bonferroni corrected *P* = 3.7 × 10^-3^; Figure 4d).

Using an electrophoretic mobility shift assay (EMSA) to examine DNA protein binding in the region, we see that, while the SNP55 risk allele causes no apparent change relative to wild-type, the SNP35 risk allele shows markedly reduced binding (Figure 4e). Three transcription factors are predicted by TRANSFAC [32] to bind the wild-type sequence but not the SNP35 variant (PRRX2, Oct-1 and Nobox; Figure S4 in Additional file 1). However, we saw no evidence that these three proteins bind the region in a supershift assay (Figure S5 in Additional file 1), suggesting other factors are critical. More than 90 transcription factors are predicted to bind the wild-type sequence using various discovery tools [33]. Thus, both SNP35 and SNP55 significantly change the silencing activity of the regulatory element, but in opposite directions and possibly through different mechanisms.

## Discussion

Through a small GWAS (fewer than 90 cases and 70 controls) we identified OCD-associated loci, which, particularly when analyzed together with regions of low variability, implicate specific cellular pathways in disease etiology. We sequenced nine of the top regions of association and five regions of fixation in eight OCD cases and eight breed-matched controls. We found a notable excess of case-only variation in evolutionarily conserved regions, particularly in non-coding elements with potential regulatory function. This suggests noncoding variation is a major factor in canine OCD similar to human neuropsychiatric diseases, and unlike most artificially induced mouse models. While the dog population is composed of >400 genetically isolated breed populations, just a small number of breeds are highly enriched for OCD, suggesting that OCD risk variants are more prevalent in these breeds. We show that the case-only variants found in the sequence data are in fact significantly more common in OCD-risk breeds compared to breeds with no increased risk of psychiatric disorders.

By comparing the sequence data using gene-based tests, we confirmed one gene (*CDH2*) and identified three novel ones (*CTNNA2*, *ATXN1*, and *PGCP*) strongly implicated for involvement in disease.

*CDH2*, a neural cadherin, encodes a calcium dependent cell-cell adhesion glycoprotein important for synapse assembly, where it mediates presynaptic to postsynaptic adhesions [34]. Disrupting expression of CDH2 in cultured mouse neurons causes synapse dysfunction, synapse elimination and axon retraction [35].

*CTNNA2* encodes a neuronal-specific catenin protein that links cadherins to the cytoskeleton [34,36] and is associated with bipolar disorder [37], schizophrenia [38], attention deficit hyperactivity disorder [38] and excitement-seeking [39]. Mice with a deletion of *CTNNA2* showed disrupted brain morphology and impaired startle modulation [40]. Cadherin-catenin complexes play a pivotal role in synapse formation and synaptic plasticity and therefore may be involved in the process of learning and memory [41].

*ATXN1* encodes a chromatin binding protein that regulates the Notch pathway [42], a developmental pathway also active in the adult brain, where it mediates neuronal migration, morphology and synaptic plasticity [43]. Mice with a deletion of *ATXN1* showed pronounced deficits in learning and memory [44].

CDH2, CTNNA2 and ATXN1 have similar spatial expression patterns in the brain and are important during brain development and for synaptic plasticity. CDH2 and CTNNA2 are highly expressed in the prefrontal cortex, amygdala, thalamus and fetal brain [34,45]. ATXN1 is highly expressed in the prefrontal cortex, basal ganglia, cerebellum and fetal brain [45,46].

Intriguingly, the three genes appear to have functional connections to the top SNPs (association *P* < 10^-5^) in a recent human OCD GWAS, which found no single associations reaching genome-wide significance, but implicated glutamatergic signaling pathways [4] (Figure S6 in Additional file 1). Most notably, one of the top associated genes in human patients, *GRIK2*, encodes a glutamate receptor recruited to the synaptic membrane by CDH2/catenin complexes [47] and another top candidate, *PKP2*, mediates CDH2 cell adhesion and desmosomal junctions [48]. In addition, several genes whose expression levels correlate with the top human OCD-associated SNPs interact with the genes we identify in dogs: LRSAM1 (cerebellum) and NARS (frontal lobe) interact with ATXN1; SPAG9 (cerebellum) acts in developmental pathways with CDH2 and CTNNA2 [49].

The fourth gene, *PGCP*, encodes a poorly characterized plasma glutamate carboxypeptidase. It may help hydrolyze N-acetylaspartylglutamate (NAAG), the third most abundant neurotransmitter in the brain, to glutamate and N-acetylaspartate (NAA) [34], suggesting a potential role in glutamatergic synapse dysfunction. *PGCP* is associated with migraine [50], which is frequently co-morbid with OCD [51].

We hypothesize that *CDH2*, *CTNNA2*, *ATXN1*, and *PGCP* may work in concert to regulate glutamatergic synapse formation and function in the cortico-striatal-thalamo-cortical (CSTC) brain circuit previously implicated in the pathogenesis of OCD [52-56].

Single variant analysis corroborates our hypothesis of dysregulated synapse formation in OCD. All four sequenced DP cases had one of two mutations (SNP35 and SNP55) in a regulatory region, between *DSC3* and *CDH2*, that we show acts as a strong silencer. The OCD-risk allele of SNP55 significantly increased the reporter gene expression while the OCD-risk allele of SNP35 had the opposite effect. While surprising, other studies have shown that either deletion or reciprocal duplication of loci such as 17p11.2 and 15q13.3 can cause neuropsychiatric disorders [57]. For SNP35, we confirmed using EMSA that the OCD-risk allele changes DNA binding. We saw no change at SNP55, although *in vitro* assays may not capture all relevant *in vivo* reactions. The regulatory element is between *CDH2* (2.2 Mb away) and *DSC3* (0.3 Mb away), both cadherin genes involved in gamma-catenin binding (Figure 1d,e), suggesting disrupted gamma-catenin binding may be an important risk factor for OCD. Additional sequence data from *DSC3* (not included in the current targeted sequencing design) and more functional analysis are needed to understand the two SNPs’ effects on *CDH2* and *DSC3*.

## Conclusions

Modeling neuropsychiatric disorders in animals is complicated by both limited understanding of the underlying neurobiology and subjective diagnostic criteria [58]. Naturally occurring canine compulsive disorder is a remarkably good model for human disease, as it is equivalent by most clinical metrics, including age of onset, symptoms, and pharmacological response. The work we present here suggests similar genetic etiology as well. We harness the limited genetic diversity of dog breeds, and high rates of OCD in particular breeds, to identify genes, pathways and non-coding candidate functional variants. Dogs suffer from a wide range of psychiatric disorders and have been strongly selected for a variety of behavioral traits, making them a uniquely powerful natural model organism for investigating inherited psychiatric diseases in humans.

## Materials and methods

### GWAS and sequencing region selection

The GWAS using the sample set and phenotypes published previously [14] was rerun using genotypes called with the new MAGIC algorithm [15]. Briefly, MAGIC (Multidimensional Analysis for Genotype Intensity Clustering) does not use prior information to make genotype calls (that is, cluster locations Hardy-Weinberg equilibrium, or complex normalization of probe intensities). Instead, it performs quantile normalization of the data for each chip independently followed by a principal component analysis of all chips on a SNP-by-SNP basis, neatly summarizing the raw data.

The processed data are then clustered into genotype calls through expectation maximization using a t-distribution mixture model. Association was calculated with a standard chi-squared test in PLINK [59] (SNP genotype rate >90%, individual genotype rate >25%, MAF >5%) and regions were defined with linkage disequilibrium -based clumping around SNPs with *P* < 0.0001 (that is, SNPs within 1 Mb with r^2^ > 0.8 and *P* < 0.01) (Table S1 and Figure S1 in Additional file 1). We identified regions of fixation as regions of >1,000 kb with more than five SNPs and >95% SNPs with MAF <0.05 and selected a subset found in breeds prone to OCD for targeted sequencing. From the associated and fixed regions we designed a 5.8 Mb targeted sequencing array that optimized inclusion of potential genes of interest within the design limitations (Tables S5 and S6 in Additional file 1).

### Gene set enrichment analysis

We expanded the GWAS regions to include all genes within 500 kb of the original region start or end (Table S1 in Additional file 1). We defined RRVs by comparing the DP breed to 24 other dog breeds from a published reference dataset and identifying the 1% least variable 150 kb regions in DP [23]. We ran INRICH with 1,000,000 permutations to test regions for enrichment in any gene sets from the GO catalog. We tested all gene sets with between 5 and 1,000 genes (downloaded from the Gene Ontology website on 18 May 2013) [17]. We used a map file of 16,433 genes lifted over to canFam2.0 from the hg19 RefSeq Gene catalog (UCSC Genome Brower, single match using default parameters) [60]. To identify gene sets with unusually high enrichment in the DP RRVs, we calculated, for all sets with *P* < 0.05 and at least 2 RRV genes in DP, the average difference in enrichment *P*-values between DP and 24 other breeds [26] (Figure 1f).

### Sequenced samples

The targeted sequencing experiment comprised a total of eight cases and eight controls from multiple breeds: DP (four cases + four controls), German shepherd (two cases + two controls), Jack Russell terrier (one case + one control) and Shetland sheepdog (one case + one control) (Figure 2a). The four DP cases supplied by Dr. Meurs showed flank sucking behavior, while the German Shepherd, Jack Russell terrier and Shetland sheepdog cases were tail-chasers.

### Targeted sequencing and variant calling

The 16 samples were individually barcoded and the targeted regions were captured by a NimbleGen Sequence Capture 385 K Array according to the manufacturer’s protocol. The captured samples were then pooled and sequenced on an Illumina Genome Analyzer II. Paired-end 76-bp reads were aligned to canFam2.0 and PCR duplicates were removed using Picard [61], and realignment and recalibration were processed through Genome Analysis Toolkit (GATK) [62,63]. SNPs and small INDELs were identified using GATK. We only considered the variants that pass the GATK standard filters. Larger structural variants were detected by GenomeSTRiP [64]. We manually checked the alignments of all discovered deletion sites for aberrant read pairs and read depth using Integrative Genomics Viewer [65] to ensure the reliability of the calls. We excluded a Shetland sheepdog pair where the control had lower SNP accuracy, when comparing case- and control-only variant counts (Figure 2b and Table S9 in Additional file 1).

### Genotyping candidate sequence variants

We first selected case-specific variants that were within evolutionarily constrained elements determined by a 29 mammals sequence dataset [27]. We then selected a subset of the variants meeting one of the following criteria: (i) case-only variants within DP breed; (ii) case-only variants within *CDH2*, *PGCP*, *CTNNA2* and *ATXN1* that are identified by gene-based analysis; (iii) case-only variants across at least two breeds; (iv) potential functional variants annotated as nonsense, splicing or missense (predicted to be ‘probably’ or ‘possibly damaging’ by Polyphen-2 [66]) and case-only variants in at least one breed; (v) variants within *CDH2* risk haplotype; and (vi) top associated variants from GWAS analysis. Of 140 variants that met one of the criteria, 127 variants passed Sequenom design standards, and were genotyped using the Sequenom iPlex system. We employed an independent set of 94 dogs that consisted of 10 dogs without obvious health problems for each of 6 OCD-risk breeds (that is, 4 sequenced breeds and West Highland white terrier (Westie) and bull terrier (bull terrier)) and two control breeds without known psychiatric problems (greyhound and Leonberger), and 14 additional OCD cases from various breeds (2 bull terrier, 2 DP, 1 German shepherd, 1 Westie, 1 Golden retriever, 1 Irish wolfhound, 1 pug, 1 Shiba, 1 Shepherd mix, 1 standard poodle, 1 Shih Tzu, 1 Welsh terrier). Genotype data were cleaned by removing samples with missing genotype rates >10% and excluding SNPs with call rates <90%. After the quality control, 114 SNPs and 88 dogs (19 (10 Leonbergers + 9 Greyhounds) from control breeds and 69 (14 cases + 5 DP + 10 bull terrier + 10 Westie + 10 German shepherd + 10 Shetland sheepdogs + 10 Jack Russell terriers) from OCD-risk breeds or cases) were retained in our analysis (Figure 2a; Table S10 in Additional file 1).

### Gene-based analysis

Each gene region was defined using the coordinates from RefSeq hg19 lifted over to canFam2.0 plus 5 kb flanking sequence on each side. We counted the number of case- and control-only variants and compared the counts for each gene. Genes that have excessive case-only variants relative to control-only variants were considered as potential risk genes for OCD. The same analysis was applied to the variants within constrained elements. To correct for gene size, we calculated the ratio of the number of case-only variants and the number of control-only variants for each gene additionally.

### Electrophoretic mobility shift assay

For each allele of the tested SNPs in a regulatory region between *CDH2* and *DSC3*, pairs of 5’-biotinylated oligonucleotides were obtained from IDT Inc. (Coralville, IA, USA; Table S13 in Additional file 1). Equal volumes of forward and reverse oligos (100 μM) were mixed and heated at 95°C for 5 minutes and then cooled to room temperature. Fifty femtomoles of annealed probes were incubated at room temperature for 30 minutes with 10 mg SK-N-BE(2) nuclear extract (Active Motif Carlsbad, CA, USA). The remaining steps followed the LightShift Chemiluminescent EMSA Kit protocol (Thermo Scientific).

### Luciferase reporter assay

The activity of a putative regulatory element and the effect of SNP35 and SNP55 on gene expression were examined by luciferase reporter assay. We PCR amplified an 879 bp-long orthologous sequence spanning SNP35 and SNP55 from human DNA samples (Table S14 in Additional file 1). The risk alleles were introduced using a site-directed mutagenesis kit. The wild-type and mutant DNA fragments were cloned into a firefly luciferase reporter plasmid (pGL4.23, Promega Madison, WI, USA). The test constructs were transiently co-transfected with a Renilla luciferase reporter plasmid (pGL4.73, Promega) as an internal control into neuroblastoma SK-N-BE(2) cells. All constructs were tested in triplicates and repeated three times in a double-blinded manner.

### Cell cultures

Human SK-N-BE(2) cells were purchased from ATCC. The cells were maintained at 37°C and 5% CO_2_ in 1:1 mixture of ATCC-formulated Eagle’s Minimum Essential Medium (EMEM) and F-12 K medium supplemented with 10% fetal bovine serum, 100 units/ml penicillin and 100 μg/ml streptomycin.

### Real-time qPCR

Real-time qPCR was performed using Quantifast SYBR Green PCR kit (QIAGEN, Hilden, Germany) on a Lightcycler 480 system (Roche Applied Science, Indianapolis, IN, USA). The reaction volumes were adjusted to 10 μl with 3 μl of DNA (10 ng), 1 μl of both primers (10 μM) and 5 μl of Master Mix. The qPCR program was as follows: pre-incubation at 95°C for 5 minutes, followed by 40 cycles of two-step amplification (10 s at 95°C, 1 minute at 60°C). All the experiments were carried out in triplicates and include negative control without DNA. The primer sets used to detect *PGCP* deletion is shown in Table S15 in Additional file 1.

### Data availability

The data presented in this publication are available through the NCBI Sequence Read Archive (SRP033723) and Gene Expression Omnibus (accession numbers GSE53488 and GSE53577). Datasets analyzed in the paper are also available at [67].

## Abbreviations

bp: base pair; DP: Doberman Pinscher; EMSA: electrophoretic mobility shift assay; GATK: Genome Analysis Toolkit; GO: Gene Ontology; GWAS: genome-wide association study; MAF: minor allele frequency; OCD: obsessive compulsive disorder; qPCR: quantitative polymerase chain reaction; RRV: regions of reduced variability; SNP: single-nucleotide polymorphism.

## Competing interests

The authors declare that they have no competing interests.

## Authors’ contributions

KLT, EKK and GF conceived and oversaw the study. EEP, JA, MD, MC and MP coordinated, collected, prepared samples and characterized samples for the study. RT, SS, MP and RS designed and performed the resequencing experiment. HJN, RT, EKK and KLT analyzed and interpreted the data. AB and AA performed genotype calling for GWAS. RT and DW performed the functional assays. HJN, RT, EKK, and KLT wrote the paper with input from the other authors. All authors read and approved the final manuscript.

## Supplementary Material

Additional file 1 Texts S1 and S2, Tables S1 to S15, and Figures S1 to S6. Click here for file
