# Supplementary material for: Candidate genes and functional noncoding variants identified in a canine model of obsessive-compulsive disorder
Source: Genome Biol. 2014 Mar 14;15(3):R25. doi: 10.1186/gb-2014-15-3-r25 (PMC4038740; doi:10.1186/gb-2014-15-3-r25)
Supplement: Additional file 1 — Texts S1 and S2, Tables S1 to S15, and Figures S1 to S6. [file gb-2014-15-3-r25-S1.pdf]

SUPPLEMENTARY INFORMATION

Text S1. Variants identified from gene-based analysis

ATXN1

ATXN1 showed a strong enrichment of case-only variants in constrained elements (10 vs. 4; **Figure 3a** and **Table S8**). Two dogs contributed to the case-only variants found in *ATXN1* (1 JR case, seven variants; 1 SS case, three variants). Of these ten case-only variants, six (five JR case variants and one SS case variants) were found in the first intron, clustering near the 5'end (**Figure 3c**). Of the remaining four, two variants were found in a SS case, one within the 3'UTR and the other in intron 3 (**Figure 3c**). The other two that were identified in a JR case failed to be aligned to hg19.

CDH2

CDH2, a gene previously associated with OCD in DP population[1], showed the strongest enrichment of case-only variants, not only in the DP samples (case-only *vs*. control-only variants, 272 *vs*. 118), but also in all the breeds together in our data set (242 *vs*. 52; **Figure 3a**). This still holds true when only considering case-specific variants within constrained elements (16 *vs*. 1; **Figure 3a**). Even if DP dogs were removed from the analysis, *CDH2* remained one of the top candidate genes (15 *vs*. 1; **Figure 3b** and **Table S8**). Taking a closer look at the variants within constrained elements in *CDH2*, we observed: (*i*) sixteen case-only variants and only one control-only variant across all the breeds; (*ii*) sixteen case-only variant and six control-only variants in DP; and (*iii*) fifteen case-only variants and no control-only variants among all the breeds excluding DP. A

cluster of the case-only variants in intron2 coincides with a strong association signal from our GWA analysis (chr7:63867472, $p=2.1\times10^{-5}$; **Figure 3c**).

CTNNA2

CTNNA2 was partially captured in our sequence data and was enriched with twelve case-only variants within constrained elements (**Figure 3a**). Two dogs, namely, a GS case (eight variants) and a JR case (five variants) contributed to the case-only variants found in *CTNNA2* region. Of twelve case-only variants within *CTNNA2*, two variants (GS case) were found in coding region, in which one variant (in exon12) was the same as the ancestral allele and the other (in exon13) as the human allele. An intronic variant from the same dog was found between these two exons. Of the remaining nine, five variants clustered around intron 8 and exon 8. Specifically, three variants (two JR case variants and one GS case variant) clustered within intron 8, and two variants (one JR case variant and one GS case variant) around exon8. The remaining four variants reside within intron 7 and intron 9, two of which reside within DNase1 hypersensitivity site (data not shown).

PGCP

PGCP was enriched with sixteen case-only variants within constrained region (**Figure 3a**). The variants were identified from three dogs, namely, a SS case (six variants), a JR case (four variants), and a GS case (six variants). Of the sixteen case-only variants, one (SS case) was found within the 3'UTR and another (JR case) in exon5, in which the identified variant was the same as the ancestral allele in other mammals. We also observed a deletion in exon2 (JRT case) and an intronic variant (GS case) near exon2

(**Figure 3c**). The remaining twelve were distributed across *PGCP*, of which three failed to liftover onto hg19.

Text S2. Coding variants

We surveyed the variants within protein-coding regions, including missense, nonsense, frame-shift and those located in essential splice sites. Six missense variants were detected in at least one case dog but not in any controls, two of which were predicted by Polyphen-2[2] to change protein function with high confidence (**Table S9**). Both of them were present in a SS case and were located inside *KIAA1530* and *calpain 14* (*CAPN14*). *KIAA153*, also known as *UV-stimulated scaffold protein A* (*UVSSA*), is widely expressed in a multiplicity of dog tissues including the brain (unpublished data). While the protein is known to interact with nucleotide excision repair complex[3], its function in the brain has not been well studied, which makes it difficult to develop a functional assay for the variant. *CAPN14* encodes the calcium-activated neutral proteinase 14 (calpain 14), which belongs to the calpain family that is involved in a variety of cellular processes including cell division, synaptic plasticity and apoptosis[4]. Its mRNA has been detected in several dog tissues including the brain (unpublished). However, when aligning the variant's flanking sequence to the human genome, a 187b-long sequence gap was present in the codon frame of the variant, making the translation of the impact of this variant into human difficult.

Supplemental Tables

Table S1. Candidate associated regions identified in GWAS

GWAS (new)		GWAS (original)		Top region (r2 > 0.8, with flanking genes)		
87 cases + 63 controls		92 cases + 68 controls				
SNP	p	SNP	p	region	size (kb)	genes
chr7 63867472	2.1E-05	chr7 63867472	7.6E-07	chr7 63741207-63954411	213	*CDH2*
chr7 61865715	1.6E-05	chr7 61835240	3.8E-06	chr7 61392736-61900158	507	*DSC3*
chr34 39694895	1.9E-07			chr34 38431782-39772222	1340	*TNIK, PLD1, TMEM212, FNDC3B, GHSR, TNFSF10*
chr26 39188777	2.1E-06			chr26 38766783-40001941	1235	*DKK1, PRKG1*
chr17 27736094	6.1E-06			chr17 27638084-27929679	292	*GALNT14, CAPN14*
chr18 56503107	1.1E-05			chr18 56127466-56771285	644	*MARK2, C11orf84, C18H11orf84, C11orf95, RTN3, ATL3, PLA2G16, HRASLS2, RARRES3, LGALS12, HRASLS5, SLC22A9, SLC22A24, SLC22A25, SLC22A10, SLC22A8, SLC22A6*
chr29 44152594	1.5E-05			chr29 43925364-44448160	523	*SDC2, PGCP, CPQ*
chr18 48909564	1.6E-05			chr18 48725507-49046739	321	*MOB2, DUSP8, KRTAP5-11, KRTAP5-2, KRTAP5-9, KRTAP5-8, KRTAP5-3, KRTAP5-4, KRTAP5-7, KRTAP5-6, IFITM10, CTSD*
chr35 18565131	1.6E-05			chr35 18409969-18872470	463	*GMPR, ATXN1*
chr18 57375179	2.4E-05			chr18 57242322-57410796	168	*ASRGL1, SCGB1D2, SCGB1D1, SCGB2A1, INCENP*
chr7 21065761	3.0E-05			chr7 20881499-22219043	1338	*EDEM3, FAM129A, RNF2, TRMT1L, SWT1, IVNS1ABP, HMCN1*
chr17 58032126	4.7E-05			chr17 57571016-58045329	474	*TRIM45, VTCN1, MAN1A2, FAM46C*
chr38 21494582	4.7E-05			chr38 20737417-22198037	1461	*LRRC52, RXRG, LMX1A, PBX1*
chr7 60990261	4.8E-05			chr7 60919881-61225006	305	*TTR, DSG2, DSG3, DSG4, DSG1*
chr35 15535554	4.8E-05			chr35 14998490-15799243	801	*PHACTR1, TBC1D7*
chr38 19918463	7.0E-05			chr38 19827135-20086439	259	*TAF1A, MIA3, AIDA, BROX, FAM177B*
chr24.29784557	8.7E-05			chr24 29224804-30560165	1335	

Table S2. Enrichment analysis of GO catalog gene sets in associated regions

TARGET	Genes in set	Genes in regions	P	Genes
GO:0045295 Gamma-catenin binding	12	3	6.9E-05	*CDH2; DSC3; DSG1*
GO:0004571 Mannosyl-oligosaccharide 1,2-alpha-mannosidase activity	9	2	0.0005	*EDEM3; MAN1A2*
GO:0030057 Desmosome	22	2	0.0015	*DSC3; DSG2,DSG3,DSG4,DSG1*
GO:0048814 Regulation of dendrite morphogenesis	13	2	0.0016	*TNIK; SDC2*
GO:0036170 Filamentous growth of a population of unicellular organisms in response to starvation	25	2	0.0020	*PLD1; MOB2*
GO:0005615 Extracellular space	748	8	0.0021	*SCGB1D2,SCGB1D1; RTN3; TTR; DKK1; BPI,LBP; TNFSF10; CPQ; CTSD*
GO:0008344 Adult locomotory behavior	41	2	0.0037	*TRMT1L; ATXN1*
GO:0001523 Retinoid metabolic process	48	2	0.0042	*TTR; SDC2*
GO:0007603 Phototransduction, visible light	50	2	0.0050	*TTR; SDC2*
GO:0043687 Post-translational protein modification	181	3	0.0056	*EDEM3; MAN1A2; GALNT14*
GO:0051286 Cell tip	5	2	0.0071	*SLC32A1; MOB2*
GO:0009267 Cellular response to starvation	48	2	0.0075	*PLD1; MOB2*
GO:0030447 Filamentous growth	56	2	0.0077	*PLD1; MOB2*
GO:0043202 Lysosomal lumen	64	2	0.0080	*SDC2; CTSD*
GO:0016339 Calcium-dependent cell-cell adhesion	25	2	0.0088	*CDH2; DSG1*

Table S3. The thirteen longest regions of fixation in the DP

chr	start	end	size	# genes	genes
3	62948826	70302993	7.35	57	*ACOX3, METTL19, TRMT44, GPR78, CPZ, HMX1, ADRA2C, LRPAP1, DOK7, HGFAC, RGS12, HTT, GRK4, NOP14, MFSD10, ADD1, SH3BP2, TNIP2, FAM193A, RNF4, ZFYVE28, MXD4, HAUS3, POLN, NAT8L, C4orf48, WHSC2, NELFA, WHSC1, LETM1, FGFR3, TACC3, TMEM129, SLBP, FAM53A, UVSSA, MAEA, FAM184B, MED28, LAP3, CLRN2, QDPR, LDB2, TAPT1, PROM1, FGFBP1, CD38, BST1, FAM200B, FBXL5, CC2D2A, C1QTNF7, CPEB2, BOD1L, BOD1L1, NKX3-2, RAB28*
3	59513417	62897146	3.38	33	*MESDC2, KIAA1199, FAM108C1, ARNT2, FAH, ZFAND6, BCL2A1, MTHFS, KIAA1024, TMED3, RASGRF1, CTSH, MORF4L1, ADAMTS7, TBC1D2B, IDH3A, ACSBG1, DNAJA4, WDR61, CRABP1, PPP2R2C, MRFAP1, S100P, BLOC1S4, KIAA0232, TBC1D14, TADA2B, GRPEL1, SORCS2, PSAPL1, AFAP1, ABLIM2, SH3TC1*
3	3548237	6087635	2.54	11	*EPB41L4A, NREP, STARD4, CAMK4, WDR36, TSLP, SLC25A46, TMEM232, MAN2A1, PJA2, FER*
24	3013164	4715848	1.70	10	*CST8, CST11, CSTL1, NAPB, GZF1, NXT1, CD93, THBD, SSTR4, FOXA2*
31	7605474	9218454	1.61	1	*GBE1*
17	45925444	47203813	1.28	2	*CTNNA2, LRRTM1*
31	3075862	4279751	1.20	6	*CGGBP1, ZNF654, HTR1F, POU1F1, CHMP2B, VGLL3*
12	36982027	38147867	1.17	2	*RIMS1, KCNQ5*
14	3737103	4832311	1.10	17	*IBA57, GJC2, GUK1, MRPL55, ARF1, WNT3A, WNT9A, PRSS38, SNAP47, OR6F1, OR13G1, OR2AK2, OR2L13, OR2L3, OR2W3, TRIM58, OR11L1*
2	62546927	63611219	1.06	13	*BBS2, OGFOD1, NUDT21, AMFR, GNAO1, CES5A, CES1, CES1P1, SLC6A2, LPCAT2, CAPNS2, MMP2, IRX6*
13	57506475	58481113	0.97	1	*TECRL*
13	6295550	7242655	0.95	6	*GRHL2, NCALD, RRM2B, UBR5, ODF1, KLF10*
11	49214235	50070536	0.86	1	*LINGO2*

Table S4. 128 regions of reduced relative heterozygosity in the DP breed

CHR	START	END	GENES
2	19440000	19590000	none
2	21780000	21990000	*MIR511-1, MIR511-2, SLC39A12*
2	80760000	80970000	*EIF4G3*
3	66030000	66210000	none
3	68820000	68970000	none
4	6660000	6870000	*EDARADD, ERO1LB, GPR137B*
4	91290000	91440000	*ANKH*
5	12930000	13140000	*OR8B2, OR8B3, OR8B4, OR8B8*
5	17160000	17370000	*PVRL1*
5	42210000	42390000	*TRIM16L, ZNF286A, ZNF287, ZNF624*
5	45690000	45930000	*KCNJ12, MAP2K3*
5	81840000	81990000	none
6	12510000	12660000	*AZGP1, COPS6, ZKSCAN1, ZNF3, ZSCAN21*
6	14100000	14250000	*BHLHA15, LMTK2, TECPR1*
6	14610000	14760000	*C7orf70, CYTH3, FAM220A, RAC1*
6	39630000	39900000	*SEPT12, ANKS3, FAM100A, GLYR1, MGRN1, ROGDI, ZNF500*
7	55320000	55530000	none
7	69300000	69450000	*ABHD3, MIB1, SNRPD1*
8	9540000	9690000	none
8	11910000	12120000	*PRKD1*
8	19680000	19860000	none
8	21060000	21240000	none
8	25110000	25320000	none
8	33330000	33480000	*CDKN3, CGRRF1, CNIH, GMFB*
8	60810000	61020000	none
8	67320000	67500000	*C14orf49, GLRX5, SNHG10*
8	70860000	71010000	*CCDC85C, CCNK, SETD3*
8	71310000	71490000	*EML1, EVL, MIR342*
9	7890000	8040000	*LLGL2, RECQL5, SAP30BP, TSEN54*
9	15450000	15720000	*BPTF, CEP95, SMURF2*
9	16110000	16320000	*PITPNC1, PSMD12*
9	18660000	18840000	*ABCA10, ABCA5, ABCA6, ABCA9*
9	51030000	51180000	*ATP2A3, CACNA1B*
10	11580000	11730000	*IRAK3, LLPH, TMBIM4*
10	31920000	32070000	*HMGXB4, ISX*
11	6270000	6420000	*AGXT2L2, COL23A1, HNRNPAB, N4BP3, NHP2, RMND5B*
11	36690000	36870000	*MPDZ*
11	38310000	38490000	*FREM1*
11	41850000	42030000	*FAM154A, HAUS6, PLIN2, RRAGA, SCARNA8*
11	52230000	52380000	none
12	14340000	14490000	*CNPY3, PTCRA, RPL7L1*
12	19500000	19650000	none
12	35490000	35640000	*COL19A1*
12	36660000	36930000	*LINC00472, MIR30A, MIR30C2, OGFRL1*
12	38220000	38370000	*KCNQ5, KHDC1, KHDC1L*
12	63780000	64050000	none
12	65940000	66120000	none
13	5880000	6030000	*ZNF706*
13	45210000	45360000	*GABRA2, GABRG1*
13	53490000	53700000	none
13	61080000	61230000	*GNRHR, UBA6*
14	48390000	48540000	*BBS9*
15	3390000	3600000	*C1orf50, ERMAP, LOC100129924, SLC2A1*
15	6870000	7110000	*MYCBP, RRAGC*
15	16290000	16440000	*CMPK1, STIL*
15	19950000	20100000	*EBNA1BP2, WDR65*
15	24450000	24600000	*SYT1*
15	28170000	28350000	none
15	33930000	34140000	none
15	40650000	40860000	none

15	53460000	53730000	*ARFIP1, TIGD4, TMEM154*
15	63900000	64050000	*TRIM61*
15	65520000	65790000	*SPOCK3*
16	56040000	56190000	none
17	18060000	18210000	*LAPTM4A, SDC1*
17	46440000	46650000	*CTNNA2*
17	65040000	65310000	*LOR, MAGI3*
18	7920000	8130000	*HPVC1*
18	15390000	15630000	*LAMB1, LAMB4, NRCAM*
18	51570000	51780000	*MYEOV*
19	13680000	13830000	none
19	21390000	21540000	*ANXA5*
19	21630000	21780000	*QRFPR*
19	23460000	23610000	*FAM123C*
19	37080000	37230000	*DPP10*
20	8580000	8790000	*C3orf25, CAND2, H1FOO, IFT122, PLXND1, RPL32*
20	16050000	16260000	*ITPR1, SETMAR, SUMF1*
20	17580000	17790000	*CNTN4*
20	20160000	20340000	none
20	23970000	24120000	*FOXP1*
20	32190000	32370000	*PTPRG*
20	33480000	33660000	*FHIT*
20	61020000	61200000	*BSG, C2CD4D, CDC34, FGF22, FSTL3, HCN2, MADCAM1, ODF3L2, POLRMT, PRSS57, RNF126, SHC2, THEG, TPGS1*
21	51570000	51780000	*KIF18A, METTL15*
22	20250000	20400000	none
22	29160000	29340000	none
22	32220000	32400000	*COMMD6, LMO7, TBC1D4, UCHL3*
22	47310000	47520000	*GPC6*
23	8850000	9000000	*ARPP21*
23	18480000	18630000	none
23	24480000	24750000	*ZNF385D*
24	3960000	4140000	*LINC00261*
24	10560000	10740000	*MACROD2*
24	44490000	44670000	*AURKA, C20orf43, CASS4, CSTF1, FAM210B, GCNT7*
25	28860000	29100000	*CTSB, DEFB131, DEFB134, DEFB135, DEFB136, FDFT1, LOC100129216, NEIL2*
25	39720000	39900000	*ATP6V1B2, SLC18A1*
25	49350000	49500000	*AGAP1*
25	53250000	53520000	*GPC1, MIR149, MYEOV2, OTOS, PP14571*
26	3630000	3780000	*GALNT9, LOC100130238*
26	4260000	4410000	*GPR133*
26	24270000	24570000	*MN1, PITPNB, TTC28, TTC28-AS1*
26	33300000	33510000	*CCDC74A, CCDC74B, KLHL22, MED15, MZT2A, MZT2B, SCARF2, SMPD4, TUBA3C*
26	33990000	34200000	*MAPK1, PPIL2, YPEL1*
26	38880000	39030000	*PRKG1*
26	40800000	41010000	*ATAD1, KLLN, PAPSS2, PTEN*
27	3780000	3960000	*GPR84, GTSF1, ITGA5, NCKAP1L, PDE1B, ZNF385A*
27	21480000	21660000	*ERGIC2, OVCH1, TMTC1*
27	37260000	37440000	*ETV6*
28	35730000	36000000	none
28	36330000	36480000	*CHST15, CPXM2*
28	39990000	40140000	none
29	19080000	19230000	*C8orf46, MYBL1, VCPIP1*
29	20910000	21060000	*C8orf34*
29	32580000	32730000	none
29	33600000	33840000	*RALYL*
30	14850000	15060000	*PLDN, SLC30A4, SQRDL*
30	22650000	22800000	*UNC13C*
30	37710000	37890000	*CT62, LRRC49, THSD4*
32	14340000	14490000	*ABCG2, PKD2*
33	12600000	12780000	none
33	33060000	33210000	*DLG1*
34	10110000	10260000	*LOC255167, NSUN2, SRD5A1, UBE2QL1*

34	16830000	17010000	*CCDC39, TTC14*
34	32670000	32820000	none
36	7200000	7350000	*CCDC148, PKP4*
36	21270000	21510000	*CIR1, GPR155, OLA1, SCRN3*
38	19470000	19740000	none
35	3240000	3510000	none

Table S5. Sequencing array design, including GWAS regions and fixed regions that overlap between the breeds predisposed with OCD.

region type	region	size (kb)	targeting strategy	total target (kb)	fraction targeted	genes targeted
GWAS	chr7 63733949-63968500	235	all	235	1.00	*CDH2*
GWAS	chr7 61598949-61916823	318	all	318	1.00	none
GWAS	chr34 38914699-39816833	902	all	902	1.00	*PLD1, TMEM212, FNDC3B, GHSR, TNFSF10*
GWAS	chr17 27631854-27926018	294	all	294	1.00	*GALNT14, CAPN14*
GWAS	chr18 56382749-56898210	515	genes & conserved elements	24	0.05	*ATL3, PLA2G16, LGALS12, HRASLS5, SLC22A8, SLC22A6, CHRM1, SLC3A2, SNHG1, SNORD30, SNORD31, SNORD22, WDR74, STX5*
GWAS	chr29 44099250-44611012	512	all	512	1.00	*PGCP, CPQ, TSPYL5*
GWAS	chr18 48718949-49010600	292	all	292	1.00	*MOB2, DUSP8, KRTAP5-11, KRTAP5-2, KRTAP5-9, KRTAP5-8, KRTAP5-3, KRTAP5-4, KRTAP5-7*
GWAS	chr35 18464094-18881027	417	all	417	1.00	*ATXN1*
GWAS	chr18 57118970-57451000	332	all	332	1.00	*AHNAK, SCGB1A1, ASRGL1, SCGB1D2, SCGB1D1, SCGB2A1, INCENP*
GWAS	chr7 20988159-21407321	419	genes & conserved elements	20	0.05	*FAM129A, RNF2, TRMT1L, SWT1, IVNS1ABP*
GWAS	chr35 15191960-15797327	605	all	605	1.00	*PHACTR1, TBC1D7*
FIXED	chr3 3485979-6250329	2764	combined*	1366	0.49	*EPB41L4A, NREP, STARD4, CAMK4, WDR36, TSLP, SLC25A46, TMEM232, MAN2A1, PJA2, FER*
FIXED	chr3 61344672-62453220	1109	genes & conserved elements	23	0.02	*PPP2R2C, MRFAP1, BLOC1S4, CNO, KIAA0232, TBC1D14, CCDC96, TADA2B, GRPEL1, SORCS2*
FIXED	chr3 64150954-65507021	1356	genes & conserved elements	120	0.09	*NOP14, C4orf10, MFSD10, ADD1, SH3BP2, TNIP2, FAM193A, RNF4, ZFYVE28, MXD4, HAUS3, POLN, NAT8L, NELFA, WHSC2, WHSC1, SCARNA22, LETM1, FGFR3, TACC3, TMEM129, SLBP, UVSSA, KIAA1530, MAEA*
FIXED	chr17 46107254-46996631	889	combined*	148	0.17	*CTNNA2, LRRTM1*
FIXED	chr19 12898854-13844607	946	combined*	158	0.17	none

** see supplementary figure 1*

Table S6. Targeted sequencing array design

CHR	START	END	TYPE	SIZE (KB)
34	38914699	39816833	GWAS	902.134
35	15191960	15797327	GWAS	605.367
29	44099250	44611012	GWAS	511.762
35	18464094	18881027	GWAS	416.933
18	57118970	57451000	GWAS	332.030
7	61598949	61916823	GWAS	317.874
17	27631854	27926018	GWAS	294.164
18	48718949	49010600	GWAS	291.651
7	63733949	63968500	GWAS	234.551
18	56469261	56472729	GWAS	3.468
18	56887849	56890630	GWAS	2.781
7	21401554	21404020	GWAS	2.466
7	21242164	21244600	GWAS	2.436
7	21396050	21398329	GWAS	2.279
7	21405399	21407321	GWAS	1.922
18	56822053	56823731	GWAS	1.678
18	56896569	56898210	GWAS	1.641
7	21236971	21238333	GWAS	1.362
18	56766349	56767618	GWAS	1.269
7	21276449	21277631	GWAS	1.182
18	56385069	56386232	GWAS	1.163
18	56465860	56466939	GWAS	1.079
7	21007671	21008728	GWAS	1.057
18	56870850	56871900	GWAS	1.050
18	56810561	56811530	GWAS	0.969
18	56764362	56765216	GWAS	0.854
7	21394299	21395123	GWAS	0.824
18	56884754	56885435	GWAS	0.681
18	56845149	56845828	GWAS	0.679
18	56754353	56755020	GWAS	0.667
7	21257973	21258622	GWAS	0.649
18	56768452	56769100	GWAS	0.648
18	56891749	56892340	GWAS	0.591
18	56418151	56418731	GWAS	0.580
18	56382749	56383236	GWAS	0.487
7	21071954	21072437	GWAS	0.483
18	56808653	56809135	GWAS	0.482
7	21273754	21274225	GWAS	0.471
7	21069049	21069507	GWAS	0.458
18	56737363	56737819	GWAS	0.456
7	20994254	20994709	GWAS	0.455
18	56488149	56488600	GWAS	0.451
7	20997879	20998328	GWAS	0.449
7	20988159	20988600	GWAS	0.441
18	56411199	56411623	GWAS	0.424
7	21014449	21014833	GWAS	0.384
18	56763254	56763629	GWAS	0.375
7	21386461	21386831	GWAS	0.370
18	56486069	56486438	GWAS	0.369
7	21281152	21281520	GWAS	0.368
18	56748854	56749222	GWAS	0.368
7	21059461	21059826	GWAS	0.365
7	21234149	21234513	GWAS	0.364
18	56425754	56426115	GWAS	0.361
7	21263454	21263800	GWAS	0.346
7	21320254	21320600	GWAS	0.346
7	20989963	20990300	GWAS	0.337
3	3624353	4029207	fixed	404.854
3	5942854	6250329	fixed	307.475

3	4800199	5084936	fixed	284.737
3	4039899	4270130	fixed	230.231
19	13514749	13656717	fixed	141.968
17	46660152	46762926	fixed	102.774
3	4593549	4695524	fixed	101.975
17	46457659	46478917	fixed	21.258
19	12898854	12906100	fixed	7.246
3	65129750	65135529	fixed	5.779
3	64154154	64159336	fixed	5.182
3	64187752	64192629	fixed	4.877
3	61779950	61783514	fixed	3.564
19	13691849	13695100	fixed	3.251
3	64987199	64990231	fixed	3.032
17	46107254	46110131	fixed	2.877
3	65118699	65121341	fixed	2.642
17	46899874	46902426	fixed	2.552
3	65494150	65496437	fixed	2.287
3	64275154	64277432	fixed	2.278
3	65003959	65006137	fixed	2.178
3	64160151	64162235	fixed	2.084
3	4368049	4370100	fixed	2.051
3	65101749	65103728	fixed	1.979
3	65187899	65189826	fixed	1.927
3	64737074	64739000	fixed	1.926
3	61938584	61940505	fixed	1.921
3	65473753	65475637	fixed	1.884
3	64280654	64282535	fixed	1.881
3	64394264	64396030	fixed	1.766
3	64767471	64769212	fixed	1.741
3	64980049	64981740	fixed	1.691
3	65476749	65478423	fixed	1.674
3	65505349	65507021	fixed	1.672
3	64543459	64545130	fixed	1.671
3	64385051	64386719	fixed	1.668
3	65213174	65214819	fixed	1.645
3	64997179	64998811	fixed	1.632
3	4356282	4357900	fixed	1.618
3	5681049	5682612	fixed	1.563
3	65158161	65159721	fixed	1.56
3	65001570	65003127	fixed	1.557
3	64197554	64199027	fixed	1.473
17	46538449	46539835	fixed	1.386
3	64587063	64588421	fixed	1.358
3	61896649	61898000	fixed	1.351
3	65007753	65009100	fixed	1.347
3	65031054	65032400	fixed	1.346
3	64436681	64438000	fixed	1.319
3	64173464	64174733	fixed	1.269
3	64351454	64352716	fixed	1.262
3	3536249	3537500	fixed	1.251
3	5470879	5472121	fixed	1.242
17	46657664	46658836	fixed	1.172
3	64597554	64598722	fixed	1.168
3	61900549	61901712	fixed	1.163
3	64926152	64927310	fixed	1.158
3	64710161	64711300	fixed	1.139
3	64179753	64180841	fixed	1.088
3	65013049	65014135	fixed	1.086
3	5227459	5228534	fixed	1.075
3	64363460	64364519	fixed	1.059
3	64831649	64832704	fixed	1.055
3	64381849	64382900	fixed	1.051
17	46578470	46579514	fixed	1.044
3	3500174	3501132	fixed	0.958

3	61823363	61824300	fixed	0.937
3	64169564	64170500	fixed	0.936
3	64164299	64165214	fixed	0.915
3	5676349	5677232	fixed	0.883
3	64823451	64824329	fixed	0.878
3	65358549	65359424	fixed	0.875
3	65486559	65487434	fixed	0.875
3	4589561	4590435	fixed	0.874
17	46896850	46897722	fixed	0.872
17	46448254	46449123	fixed	0.869
3	65112561	65113404	fixed	0.843
3	64278559	64279400	fixed	0.841
19	13696284	13697114	fixed	0.83
3	64412053	64412833	fixed	0.78
3	65484959	65485728	fixed	0.769
17	46269854	46270622	fixed	0.768
17	46607349	46608100	fixed	0.751
17	46790949	46791700	fixed	0.751
3	5559262	5560005	fixed	0.743
3	64200669	64201412	fixed	0.743
3	5177062	5177800	fixed	0.738
3	65046564	65047300	fixed	0.736
3	61796051	61796736	fixed	0.685
3	61580050	61580732	fixed	0.682
17	46995949	46996631	fixed	0.682
3	61944552	61945228	fixed	0.676
3	65458254	65458928	fixed	0.674
3	64409259	64409932	fixed	0.673
3	64928054	64928727	fixed	0.673
17	46974154	46974827	fixed	0.673
3	65447949	65448619	fixed	0.67
3	4340753	4341420	fixed	0.667
3	65327449	65328109	fixed	0.66
3	65289059	65289709	fixed	0.65
3	64982563	64983211	fixed	0.648
17	46277699	46278300	fixed	0.601
19	13326599	13327200	fixed	0.601
3	65491850	65492437	fixed	0.587
3	61912849	61913433	fixed	0.584
17	46644954	46645535	fixed	0.581
17	46236451	46237031	fixed	0.58
3	61746051	61746627	fixed	0.576
3	5640153	5640728	fixed	0.575
19	13445451	13446024	fixed	0.573
19	13834251	13834824	fixed	0.573
3	64433260	64433829	fixed	0.569
3	61344672	61345234	fixed	0.562
3	61925553	61926115	fixed	0.562
19	13070051	13070613	fixed	0.562
3	61642472	61643033	fixed	0.561
3	65325954	65326506	fixed	0.552
3	64770749	64771300	fixed	0.551
3	65203953	65204504	fixed	0.551
3	65323364	65323914	fixed	0.55
3	64392551	64393100	fixed	0.549
3	65099872	65100419	fixed	0.547
17	46568061	46568608	fixed	0.547
3	65350564	65351109	fixed	0.545
17	46924179	46924723	fixed	0.544
17	46961669	46962212	fixed	0.543
3	5548481	5549022	fixed	0.541
3	64177995	64178534	fixed	0.539
3	61556699	61557200	fixed	0.501
17	46819650	46820141	fixed	0.491

3	64283550	64284037	fixed	0.487
3	65020049	65020536	fixed	0.487
3	5243149	5243635	fixed	0.486
3	64975649	64976135	fixed	0.486
17	46583049	46583534	fixed	0.485
3	5225549	5226033	fixed	0.484
17	46855151	46855635	fixed	0.484
3	3494351	3494834	fixed	0.483
3	5280349	5280832	fixed	0.483
3	64757551	64758034	fixed	0.483
3	4343553	4344035	fixed	0.482
3	64403951	64404433	fixed	0.482
17	46872152	46872634	fixed	0.482
3	5345550	5346030	fixed	0.48
3	64681752	64682232	fixed	0.48
3	64704254	64704734	fixed	0.48
3	5637754	5638233	fixed	0.479
3	5649554	5650031	fixed	0.477
3	61391852	61392328	fixed	0.476
3	64182852	64183328	fixed	0.476
3	65373251	65373727	fixed	0.476
3	5207750	5208223	fixed	0.473
3	64284852	64285325	fixed	0.473
3	64977060	64977533	fixed	0.473
3	3518752	3519224	fixed	0.472
3	5359764	5360236	fixed	0.472
3	65210364	65210836	fixed	0.472
17	46613351	46613823	fixed	0.472
3	5773349	5773820	fixed	0.471
3	61890053	61890524	fixed	0.471
3	65481052	65481523	fixed	0.471
17	46800159	46800629	fixed	0.47
3	3495562	3496031	fixed	0.469
3	5857761	5858229	fixed	0.468
3	64218252	64218719	fixed	0.467
3	5165064	5165530	fixed	0.466
3	5304549	5305015	fixed	0.466
19	13680154	13680620	fixed	0.466
3	61552354	61552819	fixed	0.465
3	65200450	65200914	fixed	0.464
3	65343754	65344218	fixed	0.464
17	46626649	46627113	fixed	0.464
3	5903654	5904116	fixed	0.462
19	13710753	13711215	fixed	0.462
3	64168051	64168512	fixed	0.461
17	46547854	46548314	fixed	0.46
19	13267059	13267519	fixed	0.46
3	64462951	64463409	fixed	0.458
3	5408473	5408929	fixed	0.456
3	5315864	5316318	fixed	0.454
3	64781249	64781703	fixed	0.454
3	65117354	65117806	fixed	0.452
3	64862549	64863000	fixed	0.451
19	13287449	13287900	fixed	0.451
3	4393763	4394211	fixed	0.448
3	5403653	5404100	fixed	0.447
3	61367984	61368431	fixed	0.447
3	64206653	64207100	fixed	0.447
3	64692260	64692707	fixed	0.447
3	64930853	64931300	fixed	0.447
3	4337554	4338000	fixed	0.446
3	61764354	61764800	fixed	0.446
3	64758794	64759240	fixed	0.446
3	5318759	5319200	fixed	0.441

3	65148959	65149400	fixed	0.441
3	5301660	5302100	fixed	0.44
3	65022999	65023438	fixed	0.439
3	64541899	64542337	fixed	0.438
3	5667099	5667533	fixed	0.434
3	61384269	61384701	fixed	0.432
3	5327670	5328100	fixed	0.43
3	5661899	5662327	fixed	0.428
3	64779199	64779626	fixed	0.427
17	46850683	46851105	fixed	0.422
3	4374799	4375219	fixed	0.42
3	5753099	5753517	fixed	0.418
3	64213299	64213708	fixed	0.409
3	65043899	65044300	fixed	0.401
3	61788951	61789339	fixed	0.388
3	65472051	65472435	fixed	0.384
3	65483649	65484033	fixed	0.384
3	61777850	61778232	fixed	0.382
3	65000250	65000631	fixed	0.381
3	64354652	64355032	fixed	0.38
3	64421654	64422034	fixed	0.38
3	64914249	64914628	fixed	0.379
3	65017651	65018028	fixed	0.377
3	3544254	3544629	fixed	0.375
3	61859564	61859939	fixed	0.375
3	61892254	61892629	fixed	0.375
17	46876449	46876824	fixed	0.375
3	61886350	61886724	fixed	0.374
3	62438564	62438938	fixed	0.374
3	4345949	4346321	fixed	0.372
3	4380250	4380622	fixed	0.372
3	64755951	64756323	fixed	0.372
3	4366752	4367123	fixed	0.371
3	62452849	62453220	fixed	0.371
3	64556961	64557332	fixed	0.371
17	46808849	46809220	fixed	0.371
3	64601763	64602133	fixed	0.37
3	61357859	61358228	fixed	0.369
3	61389052	61389421	fixed	0.369
3	64690270	64690639	fixed	0.369
3	3572459	3572827	fixed	0.368
3	61888659	61889027	fixed	0.368
3	64559053	64559421	fixed	0.368
3	4353349	4353716	fixed	0.367
3	5400551	5400918	fixed	0.367
3	61776649	61777016	fixed	0.367
3	4376354	4376719	fixed	0.365
3	5679059	5679424	fixed	0.365
3	64911473	64911836	fixed	0.363
3	64150954	64151316	fixed	0.362
3	64427049	64427408	fixed	0.359
3	64746459	64746818	fixed	0.359
19	13049264	13049623	fixed	0.359
3	65164949	65165307	fixed	0.358
17	46781173	46781531	fixed	0.358
3	4372864	4373221	fixed	0.357
3	62221471	62221828	fixed	0.357
3	65216154	65216511	fixed	0.357
3	61394053	61394409	fixed	0.356
3	64413973	64414328	fixed	0.355
3	56197650	56198004	fixed	0.354
3	3512864	3513217	fixed	0.353
3	64166354	64166707	fixed	0.353
3	3485979	3486331	fixed	0.352

19	13797554	13797906	fixed	0.352
3	5362549	5362900	fixed	0.351
3	5754471	5754822	fixed	0.351
17	46617150	46617500	fixed	0.35
3	4592551	4592900	fixed	0.349
3	64750451	64750800	fixed	0.349
3	3552179	3552526	fixed	0.347
3	3568553	3568900	fixed	0.347
3	5245853	5246200	fixed	0.347
3	5229654	5230000	fixed	0.346
3	62315489	62315835	fixed	0.346
3	65277054	65277400	fixed	0.346
3	5235759	5236101	fixed	0.342
3	3498362	3498703	fixed	0.341
3	64978383	64978723	fixed	0.34
3	64735070	64735409	fixed	0.339
3	61798399	61798734	fixed	0.335
3	65110699	65111033	fixed	0.334
3	5311489	5311822	fixed	0.333
3	61904474	61904807	fixed	0.333
3	65128491	65128824	fixed	0.333
3	5901169	5901500	fixed	0.331
3	65016269	65016600	fixed	0.331
3	64373284	64373600	fixed	0.316
19	13844352	13844607	fixed	0.255
3	61916864	61917100	fixed	0.236

Table S7. Sequencing statistics

Sample ID	Unique Bases Aligned	On Target Bases	Mean Target Coverage	PCT of Target Bases with coverage≥2x	PCT of Target Bases with covergae≥20x
DP CASE 15237	1,139,683,488	344,896,305	69.7	99.3	90.8
DP CASE 15255	1,086,228,716	412,287,216	83.2	99.4	94.2
DP CASE 15259	1,110,107,291	429,179,317	86.5	99.5	94.1
DP CASE 15260	951,139,666	282,485,917	57.1	99.2	85.8
DP CONTROL 15227	1,154,846,994	443,969,022	89.6	99.3	94.4
DP CONTROL 15253	1,050,532,650	387,532,268	78.2	99.3	93.2
DP CONTROL 15254	1,143,518,339	436,687,467	88.0	99.5	94.1
DP CONTROL 15262	945,386,032	340,943,650	68.9	99.0	88.6
GS CASE 5913	1,133,632,267	312,071,917	63.0	99.4	91.3
GS CASE 5990	1,162,734,835	330,699,135	66.8	99.2	90.8
GS CONTROL 2722	910,366,662	385,183,461	77.7	99.5	93.5
GS CONTROL 5989	948,552,788	406,781,333	82.0	99.5	94.5
JR CASE 3094	1,267,941,716	368,157,308	74.3	99.4	92.1
JR CONTROL 205	1,062,769,474	305,843,637	61.7	99.3	89.4
SS CASE 5991	974,962,641	419,839,514	84.7	99.5	95.0
SS CONTROL 1737	945,271,757	405,413,251	81.7	99.5	94.6

Table S8. Variants detected in each individual. Variants were detected through GATK pipeline. All the samples were genotyped using Illumina CanineHD BeadChip to validate the SNP call from sequencing data.

Sample ID	SNPs	Insertions	Deletions	Variants	Ti/Tv	SNP Chip Concordance (%)
DP CASE 15237	10757	1815	2533	15105	2.58	99.0
DP CASE 15255	12211	1993	2869	17073	2.66	99.7
DP CASE 15259	10926	1839	2640	15405	2.66	99.7
DP CASE 15260	9063	1619	2254	12936	2.57	99.0
DP CONTROL 15227	11333	1902	2748	15983	2.68	99.7
DP CONTROL 15253	11399	1903	2753	16055	2.61	99.2
DP CONTROL 15254	9458	1713	2524	13695	2.57	99.5
DP CONTROL 15262	10803	1830	2672	15305	2.6	99.5
GS CASE 5913	12399	2045	2740	17184	2.56	99.7
GS CASE 5990	11330	1861	2613	15804	2.59	99.5
GS CONTROL 2722	11100	1895	2675	15670	2.57	99.7
GS CONTROL 5989	10464	1825	2648	14937	2.57	99.5
JR CASE 3094	12617	2048	2744	17409	2.62	99.2
JR CONTROL 205	11769	1929	2703	16401	2.63	97.2
SS CASE 5991	10129	1787	2599	14515	2.55	99.5
SS CONTROL 1737	11617	2061	2872	16550	2.55	93.7

Table S9. Case-/Control- Only Variants in Each Dog. All Unique Variants: the counts of variants that are present in any case but not any control for CASE sub-table and vice versa for CONTROL sub-table; Conserved Unique Variants: variants from All Unique Variants column that are within conserved elements. P-values were calculated by paired one-sided Wilcoxon signed rank test; P-value* excluded the lower-quality Shetland Sheepdog pairs. Sample names include breed as DP (Doberman pinscher), SS (Shetland Sheepdog), JR (Jack Russell terrier) and GS (German shepherd).

	All Unique Variants	Conserved Unique Variants	Conserved Unique noncoding
CASE			
DP 15237	233	12	12
DP 15259	80	6	4
DP 15255	426	19	19
DP 15260	290	15	15
SS 5991	437	27	14
JR 3094	966	47	42
GS 5913	383	14	13
GS 5990	422	26	23
CONTROL			
DP 15227	156	3	3
DP 15253	335	6	5
DP 15254	207	4	1
DP 15262	275	6	6
SS 1737	1476	41	39
JR 205	1204	42	37
GS 2722	130	4	1
GS 5989	204	1	1
P-value	0.63	0.075	0.09
P-value*	0.41	***0.018***	***0.011***

Table S10. Genotypes of 114 candidate variants on 88 dogs. SNP column indicates [chromosome.base position], A1 and A2 columns indicate reference and alternative alleles, respectively. F_A and F_U indicate the frequency of alternative alleles in OCD-enriched and control breeds, respectively.

SNP	A1	F_A	F_U	A2
chr17.46715746	A	0.007246	0	T
chr17.46791415	T	0.007246	0	C
chr18.48938780	T	0.007246	0	C
chr29.44249614	C	0.007246	0	T
chr35.18484947	G	0	0.02632	A
chr18.57136368	A	0.01449	0	T
chr29.44205914	G	0.01449	0	A
chr29.44205937	G	0.01449	0	A
chr29.44205943	A	0.01449	0	G
chr29.44306347	A	0.01449	0	G
chr35.18850625	G	0.01449	0	T
chr35.18861596	A	0.01449	0	G
chr7.63832008	G	0.02174	0	A
chr17.46897529	G	0.02174	0	A
chr29.44300177	A	0.02174	0	G
chr35.18860763	T	0.02174	0	C
chr7.63891778	T	0.02174	0.07895	C
chr18.56889625	T	0.02899	0.02632	C
chr29.44422867	G	0.04348	0	T
chr7.61693835	C	0.03623	0	T
chr7.63852056	T	0.05072	0	C
chr3.65472187	A	0.05797	0	G
chr7.61669045	A	0.05797	0.02632	T
chr7.61693952	C	0.05797	0.02632	G
chr35.18857719	A	0.05797	0.02632	T
chr19.13696914	C	0.07246	0	T
chr35.18679978	C	0.07246	0	G
chr3.64690526	A	0.07246	0.02632	G
chr7.63966490	A	0.07246	0	C
chr29.44437957	C	0.05797	0.1053	T
chr7.61728453	C	0.05072	0.1053	T
chr7.63802530	T	0.07971	0.02632	C
chr29.44306628	G	0.0942	0	A
chr7.63796858	T	0.07971	0.02632	G
chr7.63796857	A	0.07971	0.05263	C
chr7.63852467	C	0.1014	0	T

chr3.65188233	G	0.1159	0	A
chr29.44393030	G	0.1014	0	A
chr29.44397446	G	0.1232	0	T
chr7.63806661	G	0.1087	0.02632	A
chr7.63943045	A	0.1304	0	G
chr7.63950125	A	0.1304	0	G
chr35.18464093	A	0.07971	0.2105	T
chr29.44447800	T	0.08696	0.1944	C
chr7.63779775	G	0.0942	0.1316	C
chr7.63943118	A	0.1377	0	T
chr3.5470809	C	0.05797	0.3421	T
chr17.27881676	T	0.1304	0.1579	C
chr29.44180170	A	0.1765	0	C
chr3.3896965	A	0.1812	0	G
chr18.56754830	A	0.1594	0.05263	G
chr18.56768794	T	0.1739	0	C
chr3.3894924	A	0.1884	0	G
chr7.63866863	T	0.1812	0.02632	G
chr17.46781512	A	0.1884	0.02632	G
chr3.64769048	A	0.1014	0.3947	C
chr17.46791238	C	0.1449	0.2368	T
chr18.57174849	A	0.1812	0.1316	G
chr29.44338785	A	0.1667	0.1842	G
chr17.46607594	C	0.2174	0.02632	T
chr18.56737581	T	0.1522	0.2368	C
chr29.44353724	T	0.1739	0.1842	C
chr17.46478790	C	0.2029	0.1053	T
chr3.61823869	C	0.1232	0.4211	T
chr17.46781268	C	0.2391	0.02632	T
chr7.63870467	G	0.2391	0.02632	A
chr7.63870482	C	0.2391	0.02632	A
chr7.63870496	G	0.2391	0.02632	A
chr7.63870599	G	0.2391	0.02632	A
chr3.5754697	A	0.1765	0.3333	G
chr3.5471514	G	0.1739	0.3421	A
chr3.5754700	A	0.1739	0.3684	G
chr3.6063747	G	0.2029	0.3056	A
chr18.56898066	G	0.1522	0.5	A
chr29.44392979	T	0.1957	0.2632	C
chr18.48821291	T	0.2391	0.2105	C
chr7.63867146	T	0.1957	0.3947	A
chr34.39420664	T	0.2754	0.1579	G
chr29.44152594	A	0.2536	0.2632	C
chr17.46791139	C	0.1812	0.5789	G
chr18.48823350	G	0.2391	0.3684	A
chr7.63866105	A	0.2319	0.4211	C
chr17.46617340	A	0.2899	0.2778	G

chr3.65472276	A	0.2391	0.4737	G
chr17.46722666	G	0.3043	0.2632	C
chr7.63845290	T	0.3768	0	C
chr7.63857947	T	0.3551	0.1053	C
chr7.63868442	C	0.3696	0.1053	T
chr7.63912017	A	0.3986	0	G
chr29.44336600	T	0.3551	0.1579	G
chr7.63814306	A	0.3986	0.02778	G
chr7.63814172	T	0.3986	0.05263	C
chr7.63860234	A	0.3696	0.1579	G
chr29.44513249	G	0.2754	0.5789	A
chr29.44392126	G	0.2424	0.6316	A
chr7.63845160	T	0.4348	0	A
chr7.63870150	A	0.3116	0.5	G
chr7.63814541	C	0.413	0.1579	A
chr3.5559765	G	0.3623	0.4211	A
chr7.63867618	G	0.3478	0.5	C
chr7.63867879	T	0.3551	0.5	A
chr34.38986422	A	0.3551	0.5	G
chr29.44244625	G	0.4265	0.2105	A
chr7.63870805	T	0.3623	0.5	A
chr7.61865715	A	0.4348	0.2222	G
chr7.63868034	T	0.3913	0.4737	C
chr7.63867472	C	0.3913	0.5	T
chr7.63868258	T	0.3913	0.5	C
chr29.44489802	A	0.413	0.4737	C
chr3.5681788	T	0.4275	0.5526	G
chr7.63921141	C	0.5652	0.05263	T
chr34.38956076	G	0.4203	0.6053	A
chr7.63872172	G	0.4275	0.6316	C
chr34.39405162	A	0.5662	0.2368	G

Table S11. Ratio of Case/Control-Only Variants within Conserved Elements of Genes.

ALL: all dogs; DP: Doberman; OTHERS: all dogs excluding DP; Ratio: (# case-only variants) / (# control only variants+1) within a gene and its 5kb flanking.

	ALL			DP			OTHERS		
Gene	Case	Control	**Ratio**	Case	Control	**Ratio**	Case	Control	**Ratio**
CDH2	16	1	**8.0**	16	6	**2.3**	15	0	**15.0**
CTNNA2	12	2	**4.0**	0	2	**0.0**	18	2	**6.0**
ZFYVE28	3	0	**3.0**	NA	NA	**NA**	3	0	**3.0**
ATXN1	10	4	**2.0**	1	10	**0.1**	12	5	**2.0**
TMEM212	2	0	**2.0**	NA	NA	**NA**	1	0	**1.0**
CHRM1	2	0	**2.0**	NA	NA	**NA**	1	0	**1.0**
KIAA1530	2	0	**2.0**	NA	NA	**NA**	1	0	**1.0**
NOP14	2	0	**2.0**	NA	NA	**NA**	1	0	**1.0**
SLC22A8	2	0	**2.0**	2	0	**2.0**	2	0	**2.0**
PGCP	16	10	**1.5**	0	2	**0.0**	23	16	**1.4**
FNDC3B	4	2	**1.3**	0	4	**0.0**	8	1	**4.0**
CAPN14	2	1	**1.0**	NA	NA	**NA**	2	0	**2.0**
PLD1	3	2	**1.0**	0	1	**0.0**	6	2	**2.0**
HAUS3	1	0	**1.0**	NA	NA	**NA**	1	0	**1.0**
MXD4	1	0	**1.0**	NA	NA	**NA**	1	0	**1.0**
SLC22A6	1	0	**1.0**	1	0	**1.0**	1	0	**1.0**
SORCS2	1	0	**1.0**	NA	NA	**NA**	1	0	**1.0**
TADA2B	1	0	**1.0**	NA	NA	**NA**	1	0	**1.0**
TBC1D14	1	0	**1.0**	NA	NA	**NA**	1	0	**1.0**
TNIP2	1	0	**1.0**	NA	NA	**NA**	1	0	**1.0**
WDR74	1	0	**1.0**	NA	NA	**NA**	NA	NA	**NA**
GALNT14	6	6	**0.9**	0	19	**0.0**	5	2	**1.7**
PHACTR1	2	3	**0.5**	0	2	**0.0**	2	8	**0.2**
KIAA0232	1	1	**0.5**	NA	NA	**NA**	1	0	**1.0**
LRRTM1	1	1	**0.5**	NA	NA	**NA**	0	1	**0.0**
KRTAP5-8	1	2	**0.3**	1	2	**0.3**	3	0	**3.0**
C5orf13	1	2	**0.3**	3	0	**3.0**	1	5	**0.2**
CAMK4	1	2	**0.3**	1	0	**1.0**	0	4	**0.0**
AHNAK	0	1	**0.0**	1	0	**1.0**	0	1	**0.0**
DUSP8	0	1	**0.0**	2	1	**1.0**	NA	NA	**NA**
MOB2	0	1	**0.0**	NA	NA	**NA**	0	2	**0.0**
TSPYL5	0	1	**0.0**	NA	NA	**NA**	0	1	**0.0**
FAM193A	0	1	**0.0**	NA	NA	**NA**	0	1	**0.0**
MAN2A1	0	5	**0.0**	NA	NA	**NA**	0	3	**0.0**
PJA2	0	2	**0.0**	1	0	**1.0**	0	2	**0.0**
STX5	0	1	**0.0**	1	3	**0.3**	1	0	**1.0**
TMEM232	0	8	**0.0**	NA	NA	**NA**	0	8	**0.0**
WDR36	0	1	**0.0**	NA	NA	**NA**	0	1	**0.0**
HCCA2	NA	NA	**NA**	1	0	**1.0**	0	1	**0.0**
TNFSF10	NA	NA	**NA**	0	1	**0.0**	NA	NA	**NA**
EPB41L4A	NA	NA	**NA**	1	0	**1.0**	0	1	**0.0**
FER	NA	NA	**NA**	3	0	**3.0**	0	2	**0.0**
MFSD10	NA	NA	**NA**	NA	NA	**NA**	1	0	**1.0**

Table S12. Case-Only Missense Variants

Chr:position nucleotide change	dbSNP	Protein Change	Gene	Polyphen-2	Case samples
chr3:62315616 G>A		p.V40I	SORCS2	Benign	SS 5991
chr3:64757837 G>A		p.R195H	MXD4	Unknown	SS 5991
chr3:64769048 C>A		p.R404S	HAUS3	Possibly damaging	JR 3094
chr3:65472187 G>A		p.A712V	KIAA1530	Probably damaging	SS 5991
chr17:27881676 C>T		p.D620N	CAPN14	Probably damaging	SS 5991
chr18:56754830 G>A	BICF2G630690061	p.V475I	SLC22A8	Benign	DP 15259, JR 3094

Table S13. Oligonucleotide sequences (human) used for EMSA.

BIO-835.w.T.hu.F	BIO-ACCTGCACCAACTAATTAGGGTTCAAACC
BIO-835.w.T.hu.R	BIO-GGTTTGAACCCTAATTAGTTGGTGCAGGT
BIO-835.m.C.hu.F	BIO-ACCTGCACCAACTAGTTAGGGTTCAAACC
BIO-835.m.C.hu.R	BIO-GGTTTGAACCCTAACTAGTTGGTGCAGGT
BIO-855.w.A.hu.F	BIO-TCGCTGGGTGGCTCATGGACCTGCA
BIO-855.w.A.hu.R	BIO-TGCAGGTCCATGAGCCACCCAGCGA
BIO-855.m.T.hu.F	BIO-TCGCTGGGTGGCACATGGACCTGCA
BIO-855.m.T.hu.R	BIO-TGCAGGTCCATGTGCCACCCAGCGA
835.w.T.hu.F	ACCTGCACCAACTAATTAGGGTTCAAACC
835.w.T.hu.R	GGTTTGAACCCTAATTAGTTGGTGCAGGT
835.m.C.hu.F	ACCTGCACCAACTAGTTAGGGTTCAAACC
835.m.C.hu.R	GGTTTGAACCCTAACTAGTTGGTGCAGGT
855.w.A.hu.F	TCGCTGGGTGGCTCATGGACCTGCA
855.w.A.hu.R	TGCAGGTCCATGAGCCACCCAGCGA
855.m.T.hu.F	TCGCTGGGTGGCACATGGACCTGCA
855.m.T.hu.R	TGCAGGTCCATGTGCCACCCAGCGA

Table S14. Oligonucleotide sequences (human) used for luciferase assay.

enhancer_F	TTTGCTTTCTAACAATGAAACCAC
enhancer_R	TTGGCACAATTTACCGGTTT
negative_F	GAGTTGGGTGTAGGGGTCAA
negative_R	GGTTTTGAACCTCCGCAATA
enhancer_mut835_F	TGGACCTGCACCAACTAGTTAGGGTTCAAACCAAC
enhancer_mut835_R	GTTGGTTTGAACCCTAACTAGTTGGTGCAGGTCCA
enhancer_mut855_F	CTCGCTGGGTGGCACATGGACCTGCAC
enhancer_mut855_R	GTGCAGGTCCATGTGCCACCCAGCGAG
vector_F	CTAGCAAAATAGGCTGTCCC
vector_R	GACGATAGTCATGCCCCGCG

Table S15. The primer sets inside and outside the PGCP deletion used for qPCR

pgcp_del1_F	CGATCAGTGGCTTCCTTCTC
pgcp_del1_R	CGGACAATCCTGGCTTTTTA
pgcp_del2_F	CGATGACCCCATAACATTCC
pgcp_del2_R	AAGGTCTGGCTCCATCTGAA
pgcp_con1_F	GTCAGCAACAGAGCCCTTTC
pgcp_con1_R	CTCCCTCTGCTTGGAACTTG
pgcp_con2_F	AGAACACTTTGGGGCACTTG
pgcp_con2_R	GATTTCACACCCTGCTGACC

Supplemental Figures

Figure S1. Regions from GWAS used for INRICH analysis (red boxes) and targeted for sequencing (grey boxes).

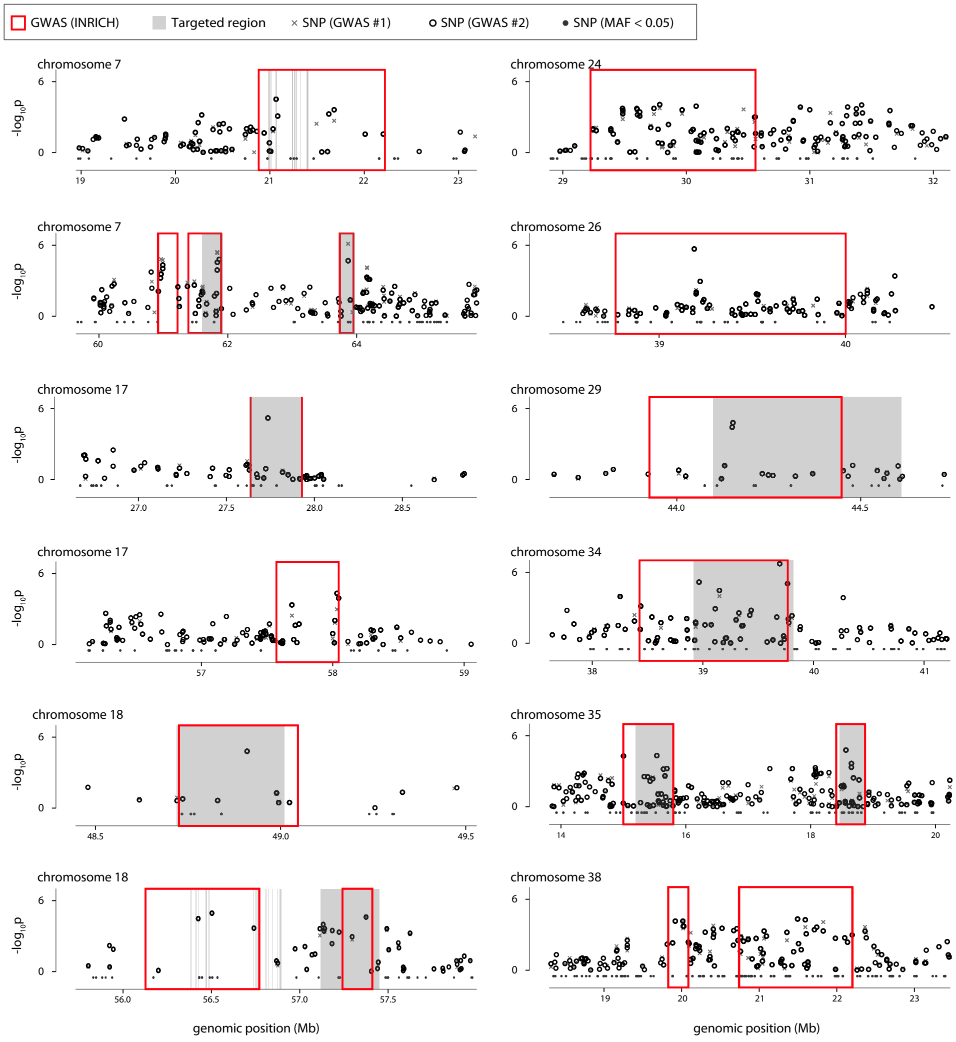

Figure S2. Pileup data in the region of 1.2kb deletion at Chr29. Pileup data for the affected Jack Russell Terrier is shown on the left, while the healthy breed-matched control is shown on the right. The top track shows the coverage of the genomic region, while the lower track shows the individual reads that are aligned to this region. Abnormal reads that may be indicative of deletion [*i.e.* reads with an insert size larger than expected] are shown in red.

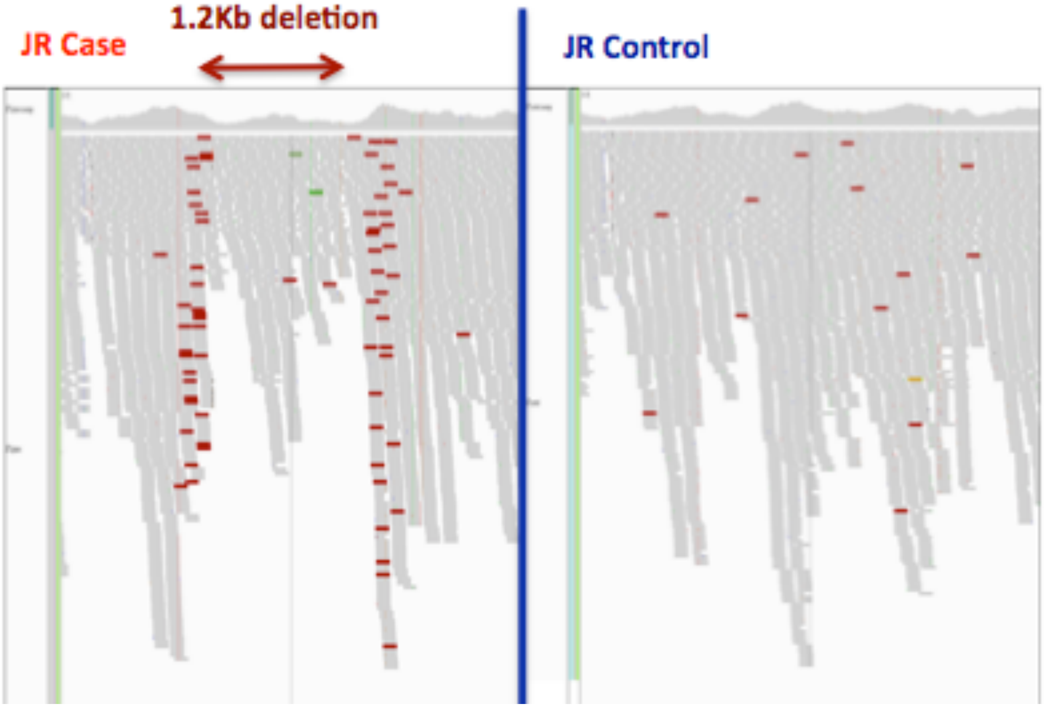

Figure S3. A 1.2kb-deletion in PGCP gene near the CCD association signal. Panel a shows -logP values for each SNP in the GWA-study in Doberman pinscher dogs. The red and green lines represent the signals from MAGIC and BRLMM algorithm, respectively. Panel b shows a 1.2kb deletion found in one Jack Russell terrier occurred in the exon2 of PGCP gene.

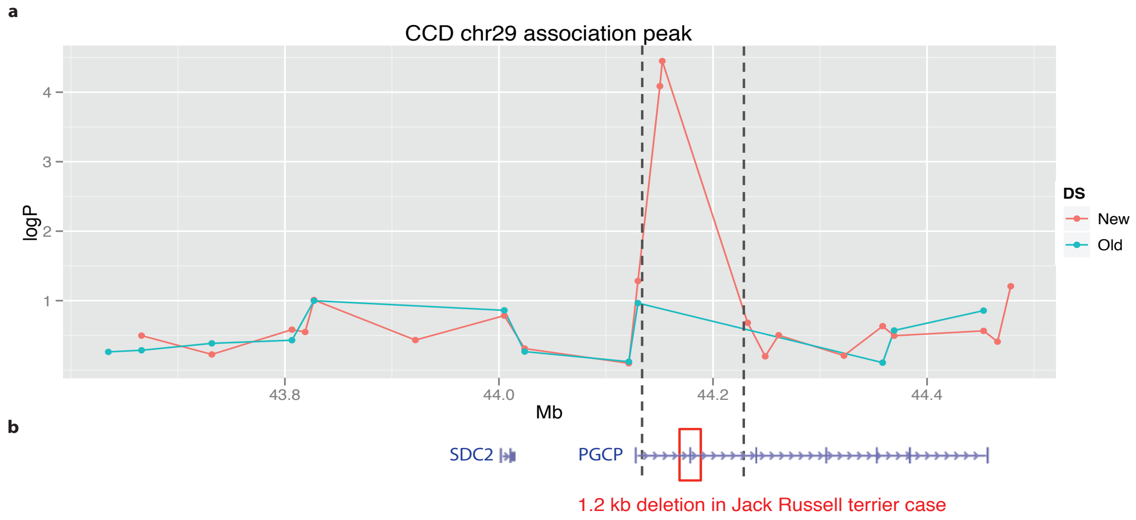

Figure S4. DNA binding motifs at chr7:61.7Mb detected by TRANSFAC

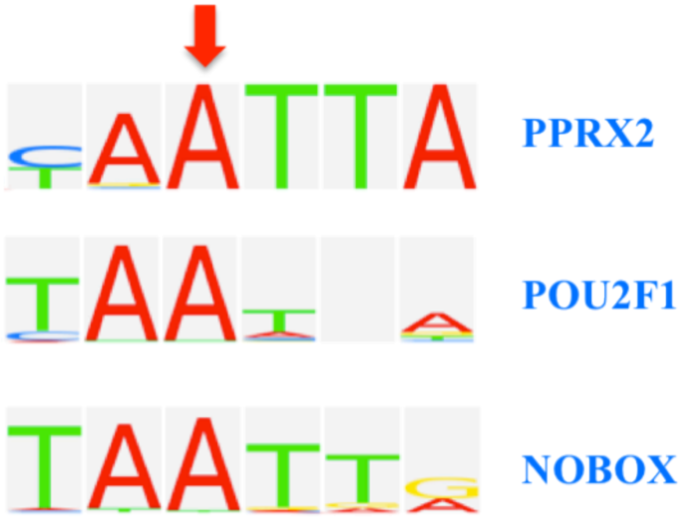

Figure S5. No DNA-protein binding difference between 855 (A) and 855 (T).

P35 wide type T allele	Gel shift				Supershift				R
Nuclear Extract	-	+	+	+	+	+	+	+	+
Labeled Oligo	+	+	+	+	+	+	+	+	+
Unlabeled competitor (200X)	-	-	+	-	-	-	-	-	-
Unlabeled cross-competitor (200X)	-	-	-	+	-	-	-	-	-
Anti-POU2F1 (10 ug)	-	-	-	-	+	-	-	-	-
Anti-POU3F2 (10 ug)	-	-	-	-	-	+	-		-
Anti-NOBOX (2.5 ug)	-	-	-	-	-	-	+	-	-
Anti-PRRX2 (10 ug)	-	-	-	-	-	-	-	+	-

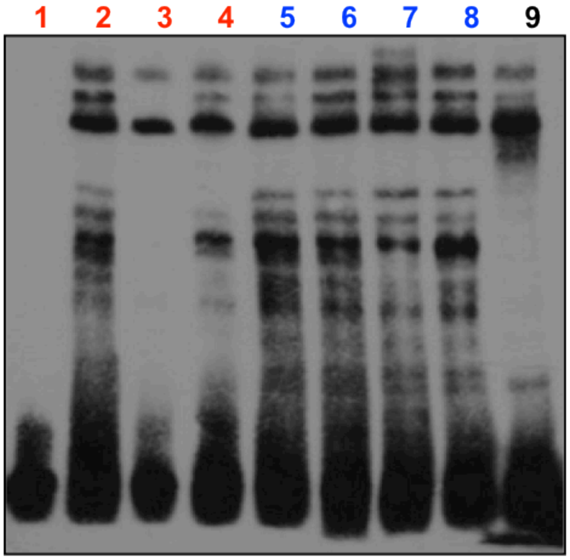

Figure S6. Functional protein association network among 115 human OCD GWAS genes and CDH2, CTNNA2, ATXN1, and PGCP that were identified from our gene-based analysis. The functional network was constructed using STRING[5]. 122 genes were extracted from Table 1 of the OCD GWAS by Stewart *et al.*[6], of which 115 genes were found in the STRING database. Note the functional connections of CDH2, CTNNA2, and ATXN1 with human OCD GWAS genes despite the generally under-connected network.

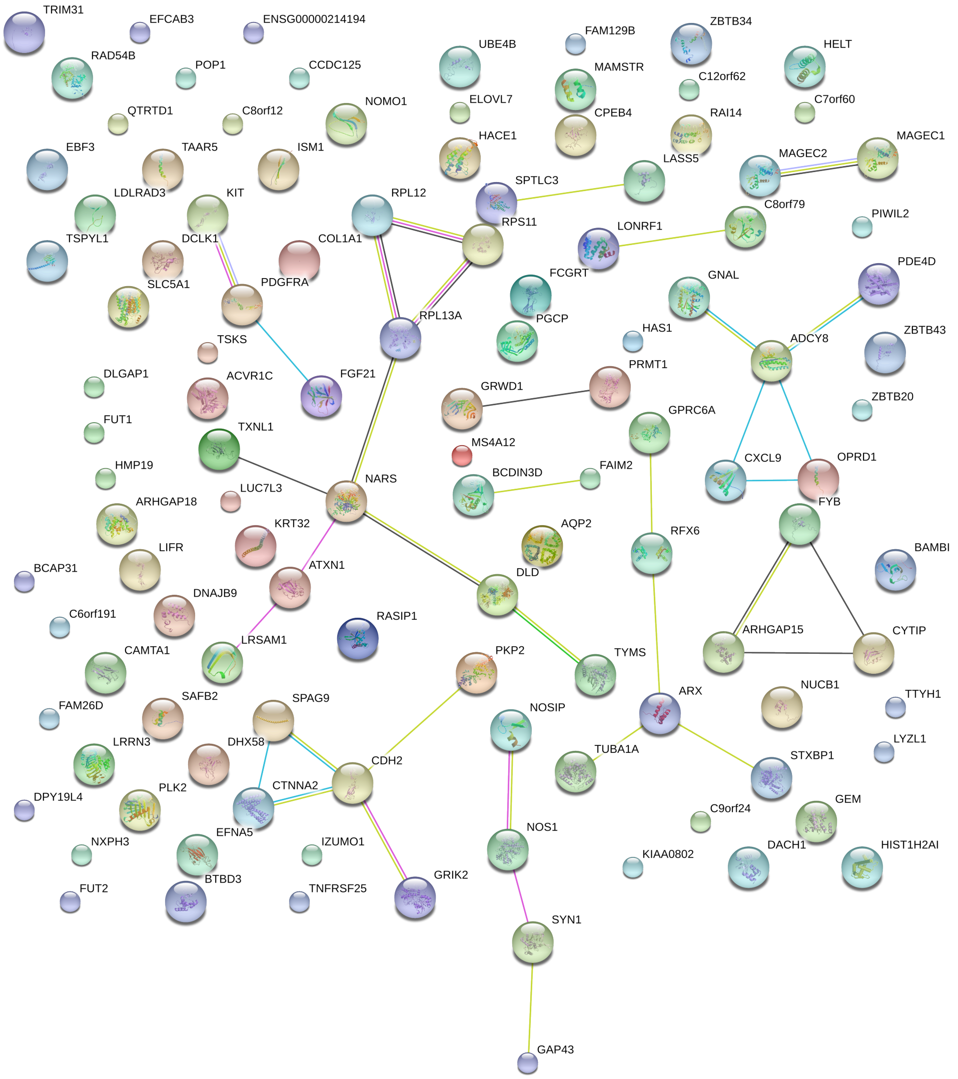

References

1. Dodman NH, Karlsson EK, Moon-Fanelli A, Galdzicka M, Perloski M, Shuster L, Lindblad-Toh K, Ginns EI: **A canine chromosome 7 locus confers compulsive disorder susceptibility.** *Molecular psychiatry* 2010, **15:**8-10.
2. Adzhubei IA, Schmidt S, Peshkin L, Ramensky VE, Gerasimova A, Bork P, Kondrashov AS, Sunyaev SR: **A method and server for predicting damaging missense mutations.** *Nature methods* 2010, **7:**248-249.
3. Sarasin A: **UVSSA and USP7: new players regulating transcription-coupled nucleotide excision repair in human cells.** *Genome medicine* 2012, **4:**44.
4. Dear TN, Meier NT, Hunn M, Boehm T: **Gene structure, chromosomal localization, and expression pattern of Capn12, a new member of the calpain large subunit gene family.** *Genomics* 2000, **68:**152-160.
5. Franceschini A, Szklarczyk D, Frankild S, Kuhn M, Simonovic M, Roth A, Lin J, Minguez P, Bork P, von Mering C, Jensen LJ: **STRING v9.1: protein-protein interaction networks, with increased coverage and integration.** *Nucleic acids research* 2013, **41:**D808-815.
6. Stewart SE, Yu D, Scharf JM, Neale BM, Fagerness JA, Mathews CA, Arnold PD, Evans PD, Gamazon ER, Osiecki L, et al: **Genome-wide association study of obsessive-compulsive disorder.** *Molecular psychiatry* 2012.
